# Optimized LC-MS/MS quantification of tuberculosis drug candidate macozinone (PBTZ169), its dearomatized Meisenheimer Complex and other metabolites, in human plasma and urine

**DOI:** 10.1016/j.jchromb.2022.123555

**Published:** 2023-01-15

**Authors:** Vincent Desfontaine, Sylvie Guinchard, Sara Marques, Anthony Vocat, Farizade Moulfi, François Versace, Jeff Huser-Pitteloud, Anton Ivanyuk, Carine Bardinet, Vadim Makarov, Olga Ryabova, Pascal André, Sylvain Prod'Hom, Haithem Chtioui, Thierry Buclin, Stewart T. Cole, Laurent Decosterd

**Affiliations:** a Laboratory & Service of Clinical Pharmacology, Department of Laboratory Medicine and Pathology, University Hospital of Lausanne and University of Lausanne, Switzerland; b Global Health Institute, School of Life Sciences, EPFL, Lausanne, Switzerland; c Innovative Medicines for Tuberculosis (IM4TB), Lausanne, Switzerland; d Federal Research Center “Fundamentals of Biotechnology RAS”, Moscow, Russia

**Keywords:** Macozinone, PBTZ169, Meisenheimer complex, MDR-tuberculosis, LC-MS/MS, Tandem mass spectrometry, Triple quadrupole, Plasma, Urine, Method validation, Human clinical trial

## Abstract

Tuberculosis, and especially multidrug-resistant tuberculosis (MDR-TB), is a major global health threat which emphasizes the need to develop new agents to improve and shorten treatment of this difficult-to-manage infectious disease. Among the new agents, macozinone (PBTZ169) is one of the most promising candidates, showing extraordinary potency *in vitro* and in murine models against drug-susceptible and drug-resistant *Mycobacterium tuberculosis*. A previous analytical method using liquid chromatography coupled to tandem mass spectrometry (LC-MS/MS) was developed by our group to support phase I clinical trials of PBTZ169. These plasma sample analyses revealed the presence of several additional metabolites among which the most prominent was H_2_PBTZ, a reduced species obtained by dearomatization of macozinone, one of the first examples of Meisenheimer Complex (MC) metabolites identified in mammals.

Identification of these new metabolites required the optimization of our original method for enhancing the selectivity between isobaric metabolites as well as for ensuring optimal stability for H_2_PBTZ analyses. Sample preparation methods were also developed for plasma and urine, followed by extensive quantitative validation in accordance with international bioanalytical method recommendations, which include selectivity, linearity, qualitative and quantitative matrix effect, trueness, precision and the establishment of accuracy profiles using β-expectation tolerance intervals for known and newer analytes.

The newly optimized methods have been applied in a subsequent Phase Ib clinical trial conducted in our University Hospital with healthy subjects. H_2_PBTZ was found to be the most abundant species circulating in plasma, underscoring the importance of measuring accurately and precisely this unprecedented metabolite. Low concentrations were found in urine for all monitored analytes, suggesting extensive metabolism before renal excretion.

## Highlights

• LC-MS/MS methods for macozinone, a drug against multi-drug-resistant tuberculosis, and metabolites in plasma and urine.
• Allows the quantification of the reduced metabolite H_2_PBTZ obtained by dearomatization of macozinone.
• H_2_PBTZ among the first examples of Meisenheimer Complex (MC) metabolites identified in mammals.
• Comprehensive stability studies of H_2_PBTZ in plasma and urine.
• Application to analyses of samples collected during Phase Ib clinical trials with healthy subjects.

## 1 Introduction

Tuberculosis (TB) is still one of the deadliest diseases worldwide [1], [2], [3], [4]. According to the 2020 WHO report on tuberculosis, approximately 10 million people fell ill with TB and 1.4 million people died from the disease in 2019, placing TB in the top 10 causes of death worldwide and the leading cause of death from a single infectious agent (ranking above HIV/AIDS) [1]. One of the main health concerns is the high proportion of multidrug-resistant TB (MDR-TB) cases, defined as resistant to rifampicin and isoniazid [1], [5], [6], [7], [8]. Worldwide in 2019, close to half a million people developed rifampicin-resistant TB and the highest incidence of MDR-TB was found in countries of the former Soviet Union. As a consequence, new drugs overcoming the resistance to usual TB treatments are urgently needed to maintain the recent decrease in TB cases over the last few years [9], [10], [11], [12], [13], [14], [15], [16].

**Fig. 1:**
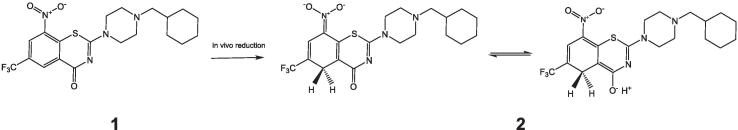
Chemical structure of macozinone (PBTZ169) subjected *in vivo* to reduction into the Meisenheimer Complex H2PBTZ, shown as the two mesomeric forms in equilibrium.

Macozinone, also known as PBTZ169 (Fig. 1, structure **1**), is a novel benzothiazinone derivative developed as a tuberculocidal candidate [17], [18], [19], [20]. It is a covalent inhibitor of decaprenylphosphoryl-beta-d-ribose 2-epimerase, a mycobacterial enzyme involved in the production of d-arabinose, a sugar essential for the biosynthesis of key cell wall components in mycobacteria [18], [21], [22], [23], [24]. This original and novel mechanism of action makes PBTZ169 an attractive candidate for the treatment of drug-susceptible and drug-resistant tuberculosis. PBTZ169 has already proven to be very effective against *Mycobacterium tuberculosis* with minimum inhibitory concentration (MIC) values below 0.0005 μg/mL [25].

PBTZ169 has successfully passed preclinical safety, toxicology and pharmacokinetic [26] assessments, and ascending dose Phase I and Phase II trials taking place both in Russia [27], and in Switzerland (study name: IM-006–11 and IM-006–13, ClinicalTrials.gov identifier: NCT03776500 and NCT03423030). An analytical method by tandem mass spectrometry was developed by our group for the quantification of macozinone and five active metabolites in plasma samples collected during the first pharmacokinetic studies performed at the Lausanne Hospital Center [28]. These plasma samples revealed the presence of additional macozinone metabolites, namely the 4-hydroxy, 4-oxo and the amino- derivatives, as well as H_2_PBTZ (Fig. 1, structure **2**, shown in mesomeric equilibrium), previously reported by Kloss *et al.* for the related benzothiazinone BTZ043 [29], resulting from the bioreductive dearomatization of PBTZ169 and which constitutes the first example of a Meisenheimer Complex (MC) metabolite identified in mammals. Moreover, H_2_PBTZ was found to be the major species in plasma. It was, therefore, important to optimize the previously developed quantification method [28], to detect the unprecedented H_2_PBTZ metabolite, as well as other newly identified metabolites, in plasma and urine analyses.

In the present paper, we have developed and extensively validated two concerted methods by liquid chromatography coupled to tandem mass spectrometry (LC-MS/MS) for the quantification of PBTZ169, H_2_PBTZ, as well as seven metabolites in human plasma and urine. These two assays have been applied to the analysis of volunteers’ samples receiving PBTZ169 during the course of multiple ascending dose Phase I trials carried out in our Center.

## 2 Materials and methods

### 2.1 Chemicals, reagents, compounds

Acetonitrile (ACN) (Lichrosolv® Reag. Ph. Eur., gradient grade for liquid chromatography), methanol (MeOH) (EMSURE® Reag. Ph. Eur. grade, for analysis) and acetic acid (100 %, LiChropur, for LC-MS) were from Merck (Darmstadt, Germany). Dimethyl sulfoxide (DMSO) was obtained from Biosolve (Valkenswaard, Netherlands). Ultrapure water was generated in-house by a Milli-Q Advantage A10 purification unit from Millipore (Bedford, MA, USA). l-ascorbic acid (99 %) was purchased form Sigma-Aldrich (Saint-Louis, MO, USA). Ammonium hydroxide solution (≥25 % in H_2_O) for LC-MS was obtained from Fluka Analytical. PBTZ169 HCl (99.9 %) was obtained from Aptuit (Oxford, United Kingdom), whereas metabolites 1OH (>99.8 %), 2OH (>99.8 %), 3OH (>99.8 %), 4OH (>99.8 %), 3-oxo (>99.1 %), 4-oxo (>99.9 %), amino (>99.5 %), H_2_PBTZ (>93 %) and internal standards PBTZ169-d11 (99.8 %, isotopic purity 98.3 %), 11526112, 11326128, 11626091, 11626092, 3-oxo-d8 (98.8 %, isotopic purity > 98 %), 4-oxo-d8 (99.5 %, isotopic purity > 98 %), Met amino-d11 (96.7 %, isotopic purity 98.3 %) and 11526102, were synthesized by the Federal Research Center “Fundamentals of Biotechnology RAS” (Moscow, Russia) (see structures of analytes in Figure S1 of the Supplementary Material). Powders were stored at −20 °C, except metabolite H_2_PBTZ which was stored at −80 °C.

### 2.2 Instrumentation

The experiments were performed using a Vanquish Flex ultra-high-performance liquid chromatography (UHPLC) system hyphenated to a TSQ Altis^TM^ triple quadrupole mass spectrometer with OptaMax NG^TM^ electrospray ionization source (ThermoFisher Scientific, San Jose, CA, USA). The Vanquish Flex was equipped with a 2-channel binary high-pressure gradient pump limited to 15′000 psi (1′000 bar), a thermostated flow-through needle autosampler with temperature range between 4 °C and 40 °C, a column oven with temperature range between 5 °C and 120 °C and a temperature-controlled (4 °C to 40 °C) charger module to increase the storage capacity of the autosampler. Data acquisition, treatment and instrument control were performed using XCalibur^TM^ version 4.2 and Chromeleon version DCMS link (ThermoFisher Scientific, San Jose, CA, USA).

### 2.3 Preparation of calibration and quality control samples

#### 2.3.1 PBTZ169, metabolites and internal standards stock solutions

An individual stock solution was prepared for each analyte and internal standard at 1 mg/mL in DMSO. After weighing and addition of DMSO, the solution was vortexed for 2 min and sonicated for 5 min to ensure total dissolution of the analyte in the solution. For all analytes except metabolite H_2_PBTZ and internal standards, the stock solution was divided into 65 µL aliquots for single use (to avoid multiple freeze–thaw cycles) and stored at −80 °C.

Because of the relative instability of metabolite H_2_PBTZ, a fresh stock solution at 1 mg/mL in DMSO was prepared *ex tempore* prior to each new set of analyses.

#### 2.3.2 Working solutions

##### 2.3.2.1 PBTZ169 and metabolites working and spiking solutions

The presence of residual PBTZ169 (∼3% w/w) in the synthetic standard of metabolite H_2_PBTZ precludes the preparation of suitable calibration samples containing both compounds simultaneously. Thus, two distinct sets of calibration (and quality control, QC) standards in human plasma or urine were systematically prepared: one for metabolite H_2_PBTZ only, and one for PBTZ169 and the seven other metabolites.

Working solution at 100 µg/mL for PBTZ169 and the seven metabolites was prepared by mixing 50 µL of each of the eight stock solutions and 100 µL of DMSO/MeOH (1:1 *v/v*). Another working solution was prepared for H_2_PBTZ alone, by mixing 50 µL of its stock solution and 450 µL of DMSO/MeOH (1:1 *v/v*).

Spiking solutions for calibration, validation standards and QC at the different concentrations were obtained by applying sequential dilutions with DMSO/MeOH (1:1 *v/v*).

##### 2.3.2.2 Internal standards working solution

Internal standards (ISTD) working solution was prepared by mixing 20 µL of PBTZ-d11, 10 µL of 11526112, 20 µL of 11326128, 40 µL of 11626091, 60 µL of 11626092, 10 µL of 3-oxo-d8, 10 µL of 4-oxo-d8, 5 µL of amino-d11 and 10 µL of 11526102 stock solutions with 4815 µL of ACN. Each individual concentration was adjusted according to the ionization efficiency of each internal standard in order to get an adequate signal intensity. This working solution was stored at −20 °C for up to 3 months.

The final internal standard solution used for urine sample dilution and plasma sample precipitation (see section 2.4) was obtained by performing a 50-fold dilution of the working solution with pure acetonitrile. The final volume was adapted according to the number of biological samples to process.

#### 2.3.3 Spiked plasma and urine samples

The validation ranges were selected to cover the clinically relevant levels expected to occur in volunteers and patients. The levels of low, medium and high QCs were 0.5, 1.5, 4, 500 and 1250 ng/mL and 5, 10, 45, 500 and 4000 ng/mL for all analytes in plasma and urine, respectively (except H_2_PBTZ in plasma: 0.5, 1, 2, 10, 1000 and 4000 ng/mL). The calibration curves were established correspondingly to encompass these QC levels, i.e. from 0.5 to 1500 ng/mL and 5 to 5000 ng/mL for all analytes in plasma and urine, respectively (except H_2_PBTZ in plasma: from 0.5 to 5000 ng/mL).

All calibration, validation and QC plasma and urine samples were obtained by spiking 25 µL of respective spiking solutions in 475 µL of blank plasma or urine, respectively. The total added volume was ≤ 10 % of the biological sample volume, thus in accordance with the recommendations for bioanalytical method validation [30]. Moreover, no protein precipitation was noticed in the presence of such a low volume of organic solvent in these calibration samples.

### 2.4 Sample preparation

#### 2.4.1 Plasma

A 150 μL-volume of acetonitrile containing internal standards was added to 50 μL of human plasma for protein precipitation. The mixture was vortexed and centrifuged at 14′000 rpm (20′160 g) for 10 min at 4 °C. A 120 μL-aliquot of limpid supernatant was directly transferred into a polypropylene (PP) HPLC vial with a conical insert from Macherey Nagel (Düren, Germany) prior to LC-MS/MS analyses.

#### 2.4.2 Urine

A generic dilute-and-shoot procedure was used to process urine samples. A 25 µL aliquot of urine sample was transferred into a 1.5 mL PP safe-lock tube and diluted with 225 µL of 20 mg/mL ascorbic acid aqueous solution and 250 µL of internal standard solution in acetonitrile. The sample was vortexed and centrifuged at 14′000 rpm (20′160 g) for 10 min at 4 °C. 150 µL of the supernatant were transferred into a PP HPLC vial with a conical insert prior to LC-MS/MS analyses.

### 2.5 Liquid chromatography-tandem mass spectrometry

#### 2.5.1 Liquid chromatography conditions

Best separation and sensitivity were obtained using a mobile phase consisting of a mixture of 2.5 mM ammonium acetate buffer at pH 5.3 and acetonitrile. The chromatographic column was an XSelect HSS T3 3.5 µm 75 × 2.1 mm from Waters (Milford, MA, USA) used with a SecurityGuard precolumn holder and Polar C18 cartridges 4 × 2.0 mm from Phenomenex (Torrance, CA, USA), both thermostated at 40 °C. The gradient program was as follows: 15 to 35 % ACN from 0 to 0.4 min, 35 to 52 % from 0.4 to 2.7 min, 52 to 95 % ACN from 2.7 to 3.5 min, isocratic step at 95 % ACN between 3.5 and 4.7 min, back to initial condition (15 % ACN) at 4.8 min and column equilibration to 6 min. The flow rate and injection volume were 0.6 mL/min and 5 µL, respectively. The injection needle was rinsed before and after drawing each sample with ACN/MeOH/water (4:4:2). The autosampler was kept at 4 °C to enhance the sample stability.

#### 2.5.2 Tandem mass spectrometry parameters

All compounds were analyzed in electrospray positive mode (ESI + ). MS/MS transitions and collision energies were determined by direct infusion of each analyte and internal standard in the mass spectrometer. Two transitions were selected for each analyte, one for quantification and one for qualitative confirmation. The summary of these parameters is shown in Table 1. Calibrated RF Lens were used for all compounds.

**Table 1:** Molecular formula and mass, MS/MS parameters and typical retention times of PBTZ169, eight metabolites and their internal standards.

Compounds	Chemical Formula	Molar mass (g/mol)	Precursor ion (*m*/*z*)	Product ion(s) (*m*/*z*)	Collision energy (V) (RCE%)	Retention time (min)
PBTZ169	C_20_H_23_F_3_N_4_O_3_S	456.5	457.3	344.0 298.0	24 (44 %) 37 (67 %)	4.16
Met 4OH	C_20_H_23_F_3_N_4_O_4_S	472.5	473.2	343.9 298.1	25 (45 %) 39 (71 %)	2.63 / 2.73
Met 3OH						2.85 / 3.00
Met 2OH						3.58
Met 1OH						3.71
Met 4-oxo	C_20_H_21_F_3_N_4_O_4_S	470.5	471.2	343.9 298.0	26 (47 %) 38 (69 %)	3.24
Met 3-oxo						3.38
Met Amino	C_20_H_25_F_3_N_4_OS	426.5	427.3	314.1 288.1	28 (51 %) 29 (53 %)	3.77
H_2_PBTZ	C_20_H_25_F_3_N_4_O_3_S	458.5	459.2	442.1	20 (36 %)	1.40
PBTZ169-d11	C_20_H_12_D_11_F_3_N_4_O_3_S	467.6	468.4	318.0	24 (44 %)	4.14
11626092	C_15_H_15_F_3_N_4_O_5_S_2_	452.4	453.1	406.9	29 (53 %)	2.76
11626091	C_16_H_15_F_3_N_4_O_5_S_2_	464.4	465.1	418.9	28 (51 %)	2.96
11326128	C_19_H_15_F_3_N_4_O_7_S_3_	564.5	565.3	344.0	30 (55 %)	3.55
11526112	C_21_H_16_F_3_N_5_O_3_S	475.5	476.1	343.9	25 (45 %)	3.67
Met 4-oxo-d8	C_20_H_13_D_8_F_3_N_4_O_4_S	478.5	479.3	351.0	26 (47 %)	3.16
Met 3-oxo-d8						3.33
Met Amino-d11	C_20_H_14_D_11_F_3_N_4_OS	437.5	438.5	314.1	26 (47 %)	3.74
11526102	C_13_H_10_N_4_O_2_	254.2	255.2	240.0	23 (42 %)	1.46

The different source parameters were assessed to reach optimal sensitivity. The spray voltage was set at 4000 V, the vaporizer and ion transfer tube temperatures were set at 350 °C, nitrogen was used for sheath and auxiliary gas at a flow of 35 and 15 arbitrary units, respectively, whereas sweep gas was not used, and the collision gas (argon) pressure was set at 2 mTorr. The mass resolution was set at 1.2 Da for each quadrupole, and the cycle time was 0.2 s. Finally, a chromatographic filter at 6 s was used and the source fragmentation was set at 0 V.

### 2.6 Bioanalytical method validation

#### 2.6.1 Selectivity and specificity

Several aspects were verified to demonstrate the selectivity and specificity of the method: (i) the absence of interference emanating from the biological matrix, (ii) assessment of ISTD to avoid interference with analytes and (iii) assessment of analytes to avoid interference with ISTD.

To this end, several analyses were performed: (i) 8 different human blank plasmas (including 6 regular and 2 lipemic) and 11 different human blank urines, all processed without ISTD, (ii) a human blank plasma and urine processed with ISTD and (iii) the highest calibration sample processed without ISTD for each biological matrix.

LC-MS/MS chromatograms were visually examined for chromatographic integrity and potential interferences.

#### 2.6.2 Matrix effect, extraction recovery and process efficiency

##### 2.6.2.1 Qualitative evaluation of matrix effect

Potential matrix effects (ME) induced by the biological matrix were qualitatively examined using the post-column strategy from Bonfiglio *et al.* [31]. Methanolic solution of all analytes and ISTD (at 100 and 500 ng/mL for ME assessment in plasma and urine, respectively) were directly infused at 10 µL/min into the MS detector while blank biological matrices (8 different human blank plasmas, including 6 regular and 2 lipemic, and 11 different human blank urines, all processed without ISTD) were injected by the LC autosampler. The obtained signals were visually examined to check for any signal perturbation (drift or shift) corresponding to ion suppression of enhancement at respective analyte’s retention time.

##### 2.6.2.2 Quantitative assessment of matrix effect, extraction recovery and process efficiency

Matrix effects (ME), extraction recoveries (ER) and process efficiencies (PE) for PBTZ169 and eight metabolites were calculated at three different concentrations (low, intermediate and high) in human plasma and urine using Matuszeswski’s strategy (10, 100 and 1000 ng/mL and 20, 500 and 2000 ng/mL, in plasma and urine, respectively) [32]. Three sets of standards were prepared: (A) analytes in neat standard solutions, i.e·H_2_O/ACN (1:3) and (1:1), for plasma and urine calculations, respectively; (B) post-extraction spiked biological matrix (8 different human plasmas, including 6 regular and 2 lipemic, and 10 different human blank urines); and (C) spiked plasma and urine processed with respective sample preparation procedure. ME was calculated using the signal area ratio of samples B/A, ER was calculated using the signal area ratio of samples C/B, and PE was calculated using the signal area ratio of samples C/A. More importantly, the internal standard-normalized ME, ER and PE (*n*-ME, *n*-ER, *n*-PE) were calculated using the analyte/ISTD peak area ratio. These values allowed verifying if the selected ISTD were suitable for correcting the potential matrix effect [33], [34].

#### 2.6.3 Trueness, precision and accuracy profiles

Trueness, precision and accuracy of the method were assessed by performing intra- and inter-assays at five concentrations (six for H_2_PBTZ in plasma), in triplicates over three different days. For plasma, the calibration ranges were 0.5–5000 ng/mL and 0.5–1500 ng/mL, and the validation samples were prepared at 0.5, 1, 2, 10, 1000 and 4000 ng/mL, and 0.5, 1.5, 4, 500 and 1250 ng/mL, for H_2_PBTZ and the remaining eight analytes, respectively. For urine, the calibration range was 5–5000 ng/mL and the validation samples were prepared at 5, 10, 45, 500, 4000 ng/mL. Each day, the concentrations of the validation standards were back-calculated using the daily calibration curve. Several regression models were evaluated to determine the one adequately describing the response concentration profile. The best regression model was selected as the one for which the deviation percentage of back-calculated concentrations from nominal values (bias) was minimal.

Trueness (systematic error) was calculated as the ratio between the average values of all back-calculated concentrations of validation standards and their nominal concentration value, at each level. Precision (random error) was evaluated thanks to the repeatability (intra-day variance) and the intermediate precision (intra-day + inter-day variances). They were reported as RSD at each concentration level [35], [36], [37].

Accuracy profiles with β-expectation tolerance interval (where β is the percentage of future results expected to lie within the interval) were constructed to depict the total error of the bioanalytical method [38], [39], [40]. For ease of interpretation, accuracy profiles were presented as total relative error (%) calculated from reference nominal concentration.

#### 2.6.4 Linearity of trueness

The linearity of the analytical method was verified by plotting calculated concentrations of validation samples in function of nominal concentrations and applying an ordinary linear square regression. The coefficient of determination r^2^ obtained for each validation day was used to attest that the method is suitable for providing proportional results.

#### 2.6.5 Limits of quantification and detection (LLOQ, ULOQ and LOD) and carryover

Based on the accuracy profiles, the lower and upper limits of quantification (LLOQ and ULOQ, respectively) were considered as the lowest and highest concentration, respectively, for which the β-expectation tolerance interval was included within the acceptance limits (±30 %) [30], [41].

The limits of detection (LOD) for each analyte were determined by injecting processed plasma or urine samples spiked with descending concentrations below their respective LLOQ and the chromatograms were visually examined to determine the LOD values (signal-to-noise ratio equal to and>3).

Carryover was assessed by injecting a blank processed biological matrix (plasma or urine) after the highest calibration standard sample.

#### 2.6.6 Integrity to dilution

Plasma and urine samples with analyte concentration values over ULOQ (i.e. 5000 ng/mL except H_2_PBTZ 10000 ng/mL) for plasma, and 10000 ng/mL for urine, were diluted 10-fold with either plasma or urine and analyzed using the methods developed to verify the possibility of such a procedure in the case of concentration result exceeding ULOQ.

The same samples were also directly analyzed without dilution. Their concentrations and deviation from the nominal value were determined through extrapolation of the calibration curve equation.

#### 2.6.7 Stabilities

##### 2.6.7.1 Short term stability in biological samples

The stability of PBTZ169 and its 8 metabolites in biological samples stored at 4 °C and at room temperature was evaluated at 10, 100 and 1000 ng/mL (10, 200 and 2000 ng/mL for H_2_PBTZ) in plasma, and 20, 500 and 2000 ng/mL in urine, by comparing the concentrations of samples analyzed after 5- and 24-hours storage against concentrations of samples measured at t_0_.

##### 2.6.7.2 Short term stability in processed samples

First, the overall stability of the analytes in processed plasma was assessed for a storage duration of 20 h at 4 °C (temperature of autosampler) to ensure the possibility to program extended injections sequences.

Secondly, the limited stability observed for metabolite H_2_PBTZ in processed urine sample has motivated the search for a suitable antioxidant to add during the sample preparation. A 25 μL aliquot of urine containing 1000 ng/mL H_2_PBTZ was diluted with 250 μL ISTD solution in ACN and 225 μL of either purified water or ascorbic acid, uric acid or dithiothreitol (DTT) aqueous solutions, all at 1 mg/mL. The evolution of H_2_PBTZ concentration in the presence of the respective antioxidant was followed in samples stored in the autosampler at 4 °C for up to 17 h. The same stability study was repeated up to 19 h using aqueous solution of ascorbic acid at 0.01, 0.5, 0.2, 1, 5, 20 and 50 mg/mL.

##### 2.6.7.3 Freeze-thaw stability

Sample stability after 3 freeze–thaw cycles from −80 °C to room temperature was verified. Spiked plasma samples were prepared at 10, 100 and 1000 ng/mL (10, 200 and 2000 ng/mL for H_2_PBTZ) and spiked urine samples were prepared at 20, 500 and 2000 ng/mL. An aliquot of each sample was either directly analyzed or subjected to 1 h-freeze/1h-thaw steps, three times prior to analysis. The mean concentration of samples after 3 freeze–thaw cycles was compared to the corresponding concentrations in freshly prepared samples.

### 2.7 Pharmacokinetics application

The two methods developed were applied to the analysis of biological samples (plasma and urine) collected during a Phase Ib randomized, placebo-controlled, multiple ascending doses clinical trial (study name: IM-006–13, ClinicalTrials.gov identifier: NCT03776500) according to a protocol approved by the Institutional Ethics Committee. Briefly, 30 healthy volunteers received various doses of PBTZ169.HCl native crystal powder (NCP) ranging from 300 to 600 mg/day for 14 days, to assess the safety and tolerability of PBTZ169. Plasma and urine samples were collected at selected time points after PBTZ169 intake on days 1 and 14 to study the pharmacokinetic profile of NCP-formulated PBTZ169 in healthy volunteers.

## 3 Results and discussion

### 3.1 Analytical method development

#### 3.1.1 LC-MS/MS method

The newly characterized metabolites of macozinone, especially the Meisenheimer Complex H_2_PBTZ, but also 4OH-, 4-oxo- and amino-PBTZ required an adaptation of our previously published analytical method [28] for the following reasons.

**Fig. 2:**
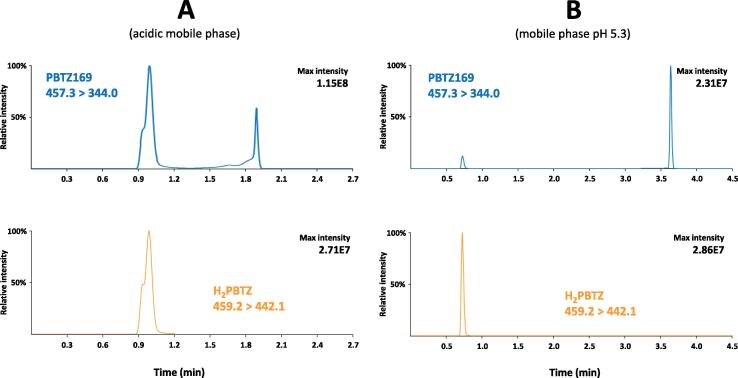
Chromatographic profiles of metabolite H_2_PBTZ synthetic powder at 5000 ng/mL in water/ACN (3:1) using a gradient mobile phase containing ACN and (A) 0.2 % formic acid or (B) ammonium acetate 2.5 mM. Blue chromatogram corresponds to PBTZ169 MS/MS transition 457.3 > 344.0 and orange chromatogram to H_2_PBTZ transition 459.2 > 442.1. (NB: the gradient program applied in this example is different from the final optimized gradient).

First, the limited stability of H_2_PBTZ necessitated a modification of the mobile phase composition. Indeed, Meisenheimer complexes are known to be unstable under acidic conditions, generally reverting to the parent molecule (here PBTZ169) [42], [43]. Therefore, formic acid, frequently added to water/acetonitrile mobile phases to improve chromatographic peaks’ shape and intensity in LC-MS, was most likely unsuitable for this particular application. This was verified by monitoring PBTZ169 transition signal (*m*/*z* 457.3 > 344.0) while injecting a 5-μl aliquot of H_2_PBTZ solution analyzed using an acidic (0.1 % formic acid) mobile phase. The observed chromatographic profile (Fig. 2**A**) is a conjunction of concurrent phenomena: i) the peak at retention time 1.88 min is due to the residual amount of PBTZ169 present in the synthetic H_2_PBTZ standard powder (∼3% w/w); ii) the peak at 0.99 min observed at PBTZ169 *m*/*z* is caused by the in-source conversion of H_2_PBTZ to PBTZ169 during the high temperature, acidic electro-spray process; iii) interestingly, the *m*/*z* signal of PBTZ169 never returns to baseline in between those two peaks. This is due to the continuous conversion of H_2_PBTZ to PBTZ169 taking place all along the chromatographic column, yielding an erratic profile, which spreads between the two peaks. Such an unusual pattern definitely impacts the peak shape of PBTZ169 precluding its accurate signal integration. Alternately, when the pH of mobile phase is increased to 5.3 using ammonium acetate buffer 2.5 mM, the profile of the chromatogram was markedly improved (Fig. 2**B**). The proportion of H_2_PBTZ reverting to PBTZ169 in the MS source is strongly decreased and the signal returns to the baseline in between the two peaks, indicating that the Meisenheimer Complex is stable during the analytical run. Hence, the sensitivity is increased for H_2_PBTZ while PBTZ169 peak is symmetrical, facilitating its integration.

**Fig. 3:**
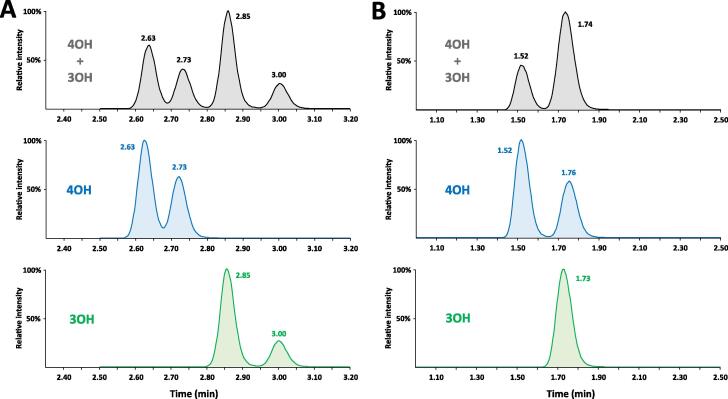
Chromatographic profiles of synthetic metabolites 4OH and 3OH at 1000 ng/mL in water/ACN (1:1) using a gradient mobile phase containing ACN and (A) ammonium acetate 2.5 mM or (B) 0.2 % formic acid in water monitored with transition 473.2 > 343.9 (metabolites OH). Black chromatogram corresponds to simultaneous injection of metabolites 4OH and 3OH, blue chromatogram to the injection of metabolite 4OH alone and green chromatogram to the injection of metabolite 3OH alone.

Second, the selectivity of the method had to be optimized because of the simultaneous quantification of several hydroxylated metabolites, namely 4OH, 3OH, 2OH and 1OH. The presence of these different isobaric compounds resulted in multiple peaks on the transition *m*/*z* 473.2 > 343.9. Generally, two peaks were observed for 4OH and 3OH, corresponding to the *cis* and *trans* diastereoisomers, according to the axial or equatorial orientation of the hydroxyl group [28]. On the other hand, a single peak was observed for both metabolites 2OH and 1OH. These two metabolites were easily separated irrespectively from the mobile phase composition. On the contrary, the separation of metabolites 4OH and 3OH was more challenging. When using an acidic mobile phase (0.1 % formic acid), one of the 4OH isomers and the two 3OH isomers co-eluted (Fig. 3**B**). These three OH isomers could not be chromatographically resolved either by altering the gradient program or by using a different stationary phase chemistry, such as pentafluorophenyl [44]. Finally, a modification of the pH of mobile phase provided a satisfactory separation of OH metabolites. Fig. 3**A** shows that all four 4OH and 3OH isomers are well resolved when using a 2.5 mM ammonium acetate buffer at pH 5.3 combined with an optimized gradient program to enhance the selectivity.

Such conditions and the gradient program described in section 2.4.2 provided satisfactory retention and separation for all analytes and were hence kept in the final optimized chromatographic method.

Finally, an individual internal standard (ISTD) was selected for each analyte in order to improve the accuracy of analytes quantitation by normalizing the differences in extraction, chromatography, ionization and detection between samples. Isotopically-labelled analogues of PBTZ169, its metabolites amino, 3-oxo and 4-oxo were available and therefore were used as internal standards. No labelled compounds were available for the other metabolites (OH and H_2_PBTZ), thus necessitating the identification of analogues suitable for quantification [45]. To this end, several molecules from the new-chemical-entities’ library of the Global Health Institute (EPFL, Lausanne, Switzerland) have been selected for their structural analogies with PBTZ169 and were injected with the optimized analytical method. Compounds with retention times close to the five analytes of interest were selected for further investigation as being the most likely to correct for any potential matrix effect. Then, a fixed concentration of the five analytes and the ISTD candidates was spiked into different blank plasma samples (n = 10). The area ratio between each analyte and its potential ISTD was monitored for each different plasma and the ISTD allowing the lowest variability of the ratio was selected. This has allowed identifying compounds 11626092, 11626091, 11326128, 11,526,221 and 11526102 as valid internal standards for metabolite 4OH, 3OH, 2OH, 1OH and H_2_PBTZ, respectively (see chemical structures in Figure S1).

#### 3.1.2 Sample preparation

##### 3.1.2.1 Plasma

Minor changes for plasma sample preparation were carried out compared to the former method developed by our group [28]. A rapid, generic protein precipitation with an organic solvent (3 to 1 organic solvent to plasma ratio) was maintained but MeOH was replaced by ACN, because ACN is a better precipitating agent than MeOH [46], [47], thereby increasing the LC column lifespan, without notable impact on analytes extraction yield and sensitivity. Finally, the optimized method gave symmetrical peaks for all analytes upon injection of up to 5 µL of supernatant into LC-MS/MS (peak distortion occurred for metabolite H_2_PBTZ at higher injection volumes). This precluded the need for a dilution step (associated with a loss of sensitivity) or the time-consuming evaporation/reconstitution procedure.

##### 3.1.2.2 Urine

Among the various sample preparation procedures described for urine samples [48], [49], the dilute-and-shoot procedure is certainly the most convenient because of its simplicity [50] and was therefore selected for our application. The dilution factor is generally chosen as a compromise between method sensitivity and potential matrix effects levelling. A 10-fold dilution factor was initially considered but intense ion suppression for early eluting compounds was observed in some concentrated urines. As isotopically-labelled internal standards were not available for all analytes, a 20-fold dilution was applied to urine for precluding any detrimental matrix effects. Sample composition after dilution was 1:1 water/ organic content for ensuring a good solubility of hydrophobic analytes while maintaining a symmetrical peak shape for all analytes with a 5-µL injection volume. The final dilution procedure consisted of the dilution of 25 µL of urine sample with 225 µL of 20 mg/mL ascorbic acid aqueous solution and 250 µL acetonitrile (containing the internal standards). Ascorbic acid was used to increase H_2_PBTZ stability in the processed sample stored at 4 °C in the autosampler rack, allowing to program longer analytical sequences (see section 3.2.7.2 for details). With such a dilution factor (1:20), all analytes could be quantified at 5 ng/mL, which was therefore selected as the lower concentration value of the validation and calibration range.

### 3.2 Bioanalytical method validation

#### 3.2.1 Selectivity and specificity

The selectivity and specificity of the developed bioanalytical methods were assessed as follows.

First, no interference was observed at the retention time of the analytes and ISTD during the injection of 8 different blank human plasmas (including 6 regular and 2 lipemic) and 11 different blank human urines, confirming that the chosen MS/MS transitions were sufficiently selective for not being perturbed by endogenous compounds (see Figure S2 of the Supplementary Material).

Second, no cross-talk interference was observed between analytes and internal standards. As shown in Figure S3 of the Supplementary Material, the only noticeable interference was the two peaks of Met 2OH and 1OH on the transition of internal standard 11,526,112 due to isotopic contribution M + 3 (molecular mass of 473 and 476 for precursor ions of Met OH and 11526112, respectively). However, the 3 peaks were chromatographically separated (retention time of 11,526,112 was 3.67 min, just between the two peaks of Met 2OH et 1OH, 3.58 and 3.71 min respectively) and, thus, this interference was not detrimental to the analyses.

#### 3.2.2 Matrix effect, extraction recovery and process efficiency

##### 3.2.2.1 Qualitative evaluation of matrix effect

The profiles obtained during the qualitative matrix effects assessment in human plasma and urine samples are shown in Fig. S4A and B, respectively. In urine, no major perturbations were observed at the analytes’ retention times, indicating negligible impact of urine matrix on target analytes ionization. On the other hand, a major zone of signal perturbation was identified for plasma samples between 3.6 and 4.2 min. This was most probably due to the elution of phospholipids present in plasma. Three analytes, namely PBTZ169, 1OH and amino metabolites eluted during this period and could potentially be affected by this matrix effect, underscoring the need for internal standards with close retention time to these three analytes. The impact of phospholipids on ionization was examined in detail during the quantitative matrix effect assessment (see below).

**Fig. 4:**
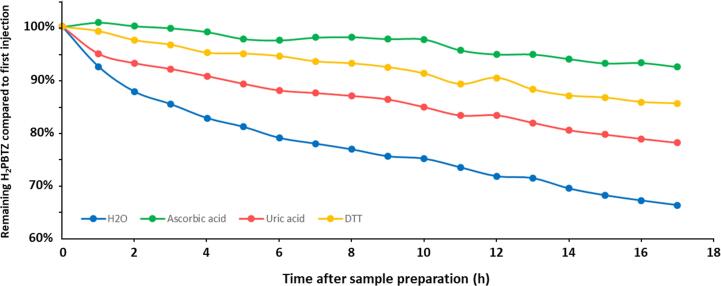
Evolution of H_2_PBTZ/ISTD 11526102 peak area ratio over time in human urine processed with different antioxidants and kept at 4 °C in the autosampler. 25 μL of human urine containing 1000 ng/mL H_2_PBTZ were diluted with 250 μL ISTD solution in ACN and 225 μL of either purified H_2_O (blue), aqueous ascorbic acid (green), uric acid (red) or DTT (yellow) solutions, all at 1 mg/mL.

##### 3.2.2.2 Quantitative assessment of matrix effect, extraction recovery and process efficiency

The results of the quantitative determination of matrix effects in human plasma and urine, carried out with the Matuszewski’s approach, are reported in Tables S1 and S2, respectively. For urine, mean values for normalized matrix effects, recoveries and process efficiencies for all analytes at 20, 500 and 2000 ng/mL were comprised between 85 and 115 %, while precision (RSD) values were lower than 15 %. In conclusion, the selected internal standards were suitable to correct appropriately any signal variability in urine matrix and the potential loss of analytes during sample preparation. For plasma, the same observation was made for PBTZ169 and metabolites 4OH, 3OH, 4-oxo, 3-oxo and H_2_PBTZ at 10, 100 and 1′000 ng/mL (10, 200 and 2′000 ng/mL for H_2_PBTZ). On the other hand, for metabolite 2OH, a slight matrix effect was observed (+20 %) at the lowest concentration, which was however deemed acceptable. For metabolite 1OH, a significant negative matrix effect (ion suppression) of −40 % was observed, albeit with limited variability. This was due to: (i) this metabolite co-eluted with phospholipids (as already explained in section 3.2.2.1), and (ii) the internal standard used is only a close analogue (i.e. not an isotopically labelled molecule). Yet, plasma matrix-matched calibration was always used, and given the small matrix effect variability, should compensate for most of the matrix effect. More surprisingly, an unexpected positive matrix effect (+40 %) was observed for the amino metabolite despite the use of an isotopically labelled internal standard. Yet, given its limited variability (RSD within 4–6 %), it can adequately be circumvented, as for metabolite 1OH, by the matrix-matched calibration strategy.

#### 3.2.3 Trueness, precision and accuracy profiles

For all analytes in human plasma and urine, the best validation results were obtained when using the quadratic logarithmic regression model for the response function. These results are reported in Table 2, Table 3 for the validation in human plasma and urine, respectively. For all analytes in plasma, trueness, repeatability, and intermediate precision were comprised within 95.9–106.3 %, 0.7–8.2 % and 2.0–8.8 %, respectively. For urine, they were comprised within 97.8–105.4 %, 2.4–8.8 % and 2.9–8.8 %, respectively. All results are thus well in line with the FDA recommendations [30].

**Table 2:** Trueness and precision results obtained for the validation in human plasma.

**Analytes**	**Concentration levels of validation samples** (ng/mL)	**Trueness** (%)	**Precision**	
			Repeatability (%)	Intermediate precision (%)
PBTZ169	0.5	102.5	5.9	5.9
	1.5	96.2	4.0	5.0
	4	98.9	2.4	2.7
	500	103.0	3.7	4.3
	1250	100.0	2.3	2.6
Met 4OH	0.5	101.7	2.3	4.3
	1.5	102.3	1.4	7.7
	4	102.6	2.4	7.2
	500	105.3	1.3	5.9
	1250	101.3	2.7	4.0
Met 3OH	0.5	101.3	3.2	5.5
	1.5	100.0	3.4	5.8
	4	102.4	1.6	7.2
	500	104.5	0.9	4.7
	1250	101.0	2.9	3.3
Met 2OH	0.5	98.9	2.7	4.3
	1.5	96.3	2.5	5.5
	4	101.5	3.3	5.8
	500	104.0	2.4	5.4
	1250	100.3	3.1	3.1
Met 1OH	0.5	101.2	3.5	5.2
	1.5	98.3	3.4	4.0
	4	98.2	2.8	4.7
	500	101.8	2.1	3.5
	1250	100.8	3.6	3.6
Met 4-oxo	0.5	103.3	5.5	7.0
	1.5	101.5	2.2	8.3
	4	103.0	1.6	8.1
	500	104.8	0.7	6.1
	1250	101.9	1.8	4.7
Met 3-oxo	0.5	104.5	5.7	6.3
	1.5	100.6	2.0	5.1
	4	101.7	2.7	6.7
	500	103.6	1.3	5.8
	1250	100.6	2.0	3.8
Met Amino	0.5	101.3	8.2	8.8
	1.5	95.9	7.0	7.0
	4	97.4	7.0	7.0
	500	106.3	5.7	5.7
	1250	97.3	4.9	4.9
H_2_PBTZ	0.5	101.9	3.2	3.8
	1	99.4	4.0	4.0
	2	100.7	3.4	3.4
	10	97.0	2.6	2.6
	1000	99.5	1.5	6.1
	4000	98.6	1.7	2.0

**Table 3:** Trueness and precision results obtained for the validation in human urine.

**Analytes**	**Concentration levels of validation samples** (ng/mL)	**Trueness** (%)	**Precision**	
			Repeatability (%)	Intermediate precision (%)
PBTZ169	5	101.4	6.0	6.8
	10	100.9	8.2	8.2
	45	99.3	5.3	5.3
	500	101.9	3.6	4.9
	4000	100.2	3.8	3.8
Met 4OH	5	100.4	2.4	3.7
	10	101.9	7.8	7.8
	45	99.5	4.3	4.3
	500	102.2	3.8	3.8
	4000	99.9	3.6	3.6
Met 3OH	5	99.4	2.7	4.6
	10	99.7	8.3	8.3
	45	100.0	3.7	3.7
	500	102.5	4.2	4.2
	4000	99.9	3.1	3.1
Met 2OH	5	100.3	3.4	3.7
	10	100.7	8.0	8.0
	45	100.4	6.6	6.6
	500	98.8	3.7	5.3
	4000	99.2	4.8	5.0
Met 1OH	5	98.2	4.4	4.4
	10	100.9	5.8	7.9
	45	99.6	4.4	4.9
	500	103.3	4.5	4.5
	4000	105.4	5.2	5.2
Met 4-oxo	5	101.7	4.1	4.1
	10	103.6	4.6	4.6
	45	99.7	2.9	2.9
	500	104.4	4.6	4.6
	4000	100.6	3.3	3.7
Met 3-oxo	5	99.8	3.3	3.3
	10	102.1	7.4	7.4
	45	99.2	3.4	3.4
	500	104.8	4.5	4.5
	4000	99.7	3.3	3.3
Met Amino	5	98.4	4.9	4.9
	10	100.1	8.8	8.8
	45	97.8	3.6	3.8
	500	100.5	4.7	4.7
	4000	100.6	3.9	4.7
H_2_PBTZ	5	102.7	6.9	8.7
	10	99.0	6.2	6.2
	45	101.0	5.6	5.6
	500	101.8	4.9	5.0
	4000	99.1	4.5	4.9

Accuracy profiles with β-ETIs (Expectation Tolerance Intervals) were established using a β value of 90 %. As shown in Figures S5 and S6 for human plasma and urine, respectively, all accuracy profiles were within the acceptance limits recommended for bioanalytical method validation (±30 %) [41].

#### 3.2.4 Linearity of trueness

The graphical plots established for the verification of the method linearity are shown in Figures S7 and S8, for human plasma and urine, respectively. The results were considered satisfactory for PBTZ169 and all metabolites as all determination coefficients values were > 0.99.

#### 3.2.5 Limits of quantification and detection (LLOQ, ULOQ and LOD) and carryover

As defined by the lower and the upper validation standards samples concentrations having acceptable accuracy and precision values, the LLOQ was 0.5 ng/mL for all analytes in human plasma and the ULOQ was 1250 ng/mL (4000 ng/mL for H_2_PBTZ). In human urine, the LLOQ and ULOQ were 5 and 5000 ng/mL, respectively.

Plasma and urine samples spiked with descending concentrations below the respective LLOQ were injected using the developed method in order to determine the LOD for each analyte. The LOD values obtained for human plasma and urine are summarized in Table S3 with the corresponding chromatograms shown in Figures S9 and S10 for plasma and urine, respectively.

Carryover for the plasma and urine methods was assessed by injecting a blank processed matrix sample (plasma or urine) after the highest calibration standard sample. In both matrices, no relevant carryover issue was observed for metabolites 4OH, 3OH, 4-oxo, 3-oxo and H_2_PBTZ. For the other analytes, a slight carryover effect was observed but could be reduced to acceptable levels for those analytes, by one and two blank 5-μl injections of ACN/H2O (3:1) after the highest calibration sample in urine and plasma, respectively (see Table S4).

#### 3.2.6 Integrity to dilution

In case of concentrations exceeding ULOQ values, two approaches were compared, namely: (i) dilution of the sample with blank matrix before regular analysis, and (ii) extrapolation of the regression equation beyond the validated range. The results of these experiments are reported in Tables S5 and S6 for plasma and urine, respectively. In urine, both strategies gave reliability for all analytes concentrations deviating less than 15 % from nominal values with a precision value lower than 6 %. In plasma, the dilution strategy gave a bias for the two most hydrophobic analytes PBTZ169 and its amino metabolite, with a recovery of 76 and 85 %, respectively. This is probably due to adsorption issues on the plastic component of Eppendorf tubes used to perform the 10-fold dilution in plasma. Alternately, extrapolated results from undiluted samples obtained with the usual regression models deviated less than 15 % from nominal values (with a precision value lower than 5 %) for all analytes, except metabolite amino. In conclusion, the dilution strategy should be used for the amino metabolite whereas the extrapolation is advisable for PBTZ169. For all other analytes, both strategies were acceptable.

#### 3.2.7 Stabilities

##### 3.2.7.1 Short-term stability in biological samples

Stability results for stability in human plasma and urine samples are reported in Tables S7 and S8, respectively.

Only PBTZ169, amino metabolite and, unexpectedly, H_2_PBTZ were stable in plasma at room temperature and 4 °C for 24 h. Metabolite 1OH was only stable at 4 °C when stored for 24 h. Regarding the other metabolites, deviation from the initial value exceeded −15 % even after only 5 h at 4 °C. This indicates that collected plasma samples must be stored at −20 °C or preferably at −80 °C without delay after blood collection and centrifugation.

In urine, while the amino metabolite was found stable at room temperature and 4 °C during 24 h, severe degradation was observed for PBTZ169 and the sother metabolites, especially after 24 h. Such degradation appears concentration-dependent with higher concentrations showing longer sample stability. In conclusion, urine samples have to be stored at −20 °C or preferably at −80 °C without delay after collection.

##### 3.2.7.2 Short-term stability in processed samples

The overall stability of PBTZ169 and eight metabolites in processed plasma was assessed for a storage duration of 20 h at 4 °C (i.e. temperature of the autosampler). The results, presented in Table S9, show that all the analytes, also notably H_2_PBTZ, are stable at least 20 h.

**Fig. 5:**
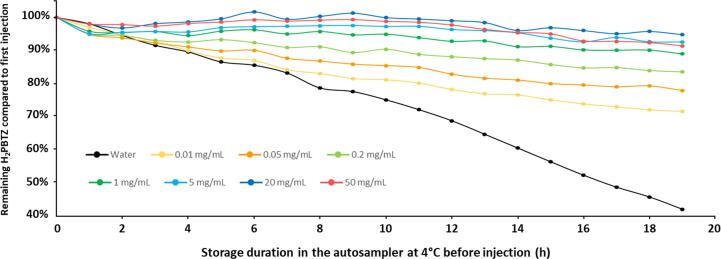
Evolution of H_2_PBTZ/ISTD 11526102 peak area ratio over time in human urine processed with different ascorbic acid concentrations and kept at 4 °C in the autosampler. 25 μL of human urine containing 1000 ng/mL H_2_PBTZ were diluted with 250 μL ISTD solution in ACN and 225 μL of aqueous ascorbic acid solutions at different concentrations or pure water (see legend).

Unlike in plasma, H_2_PBTZ was not stable in processed urine. To improve its stability (i.e. with the possibility of extended analytical sequences) the antioxidants ascorbic acid, uric acid or dithiothreitol (DTT) have been individually added during the sample preparation procedure and the remaining level of H_2_PBTZ was monitored in samples stored during an extended time in the autosampler at 4 °C. The evolution of H_2_PBTZ levels using the respective antioxidant solutions or purified H_2_O, is shown in Fig. 4. The three antioxidants improved the stability of H_2_PBTZ, with the highest performance shown for ascorbic acid, which was selected for further investigation. Fig. 5 shows the same stability experiment using ascorbic acid at 0.01, 0.05, 0.2, 1, 5, 20 and 50 mg/mL in the sample preparation. H_2_PBTZ stability could be improved in the presence of ascorbic acid at concentrations from 0.01 to 5 mg/mL, and remains essentially constant at ascorbic acid above 5 mg/mL. Ascorbic acid at 20 mg/mL was thus selected for the final sample preparation procedure (highest curve on Fig. 5). At such ascorbic acid concentration, subsequent stability experiments have indicated that all analytes were stable in urine extract samples for at least 20 h in the autosampler at 4 °C (see Table S10).

##### 3.2.7.3 Freeze-thaw stability

The results for the analytes stability after three freeze/thaw cycles from −80 °C to room temperature are presented in Table S11. All the calculated recoveries compared to freshly prepared samples were between 92 and 106 %, indicating that PBTZ169 and all the metabolites (including H_2_PBTZ) were stable in both human plasma and urine after the three freeze/thaw cycles.

### 3.3 Pharmacokinetics application

The two methods were used in the clinical trial described in section 2.7.

**Fig. 6:**
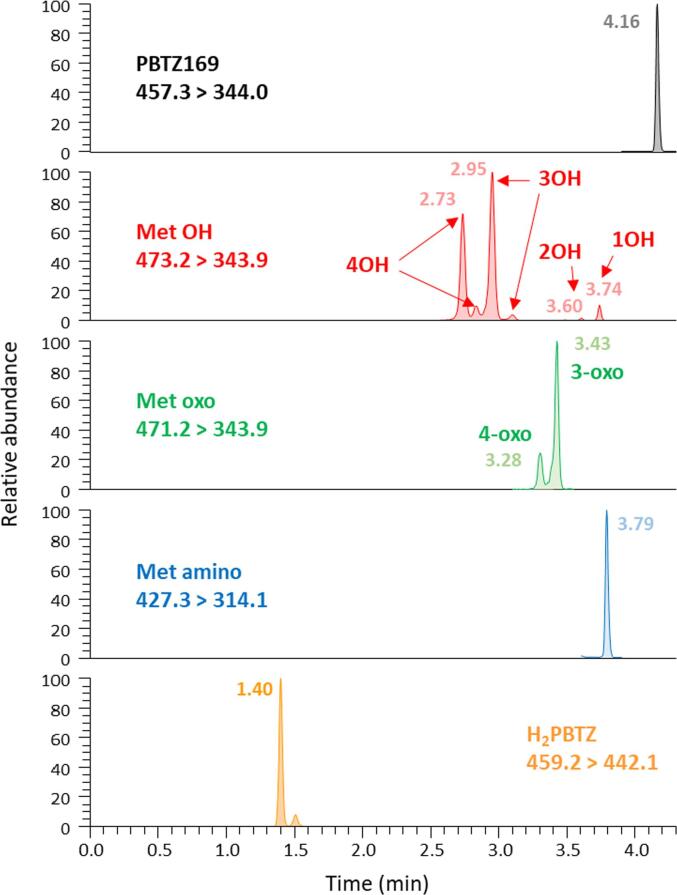
Chromatographic profile of a healthy volunteer’s plasma sample, 30 min after the first intake of 300 mg PBTZ169.HCl on day 1 of the clinical trial (300 mg twice a day during 14 days).

The principal species in plasma are PBTZ169, H_2_PBTZ, followed by the metabolites 3OH and 4OH. Alternately, metabolites 2OH, 1OH and 4-oxo were generally absent or observed at very low concentrations. Fig. 6 shows the chromatographic profile of a healthy volunteer’s plasma sample collected 30 min after the first intake of 300 mg PBTZ169.HCl. In this plasma sample, the concentrations of PBTZ169, H_2_PBTZ, metabolites 3OH, 4OH, 3-oxo, amino, 4-oxo and 1OH were 177.5, 246.1, 93.2, 67.4, 11.1, 4.3, 3.2 and 1.6 ng/mL, respectively. Metabolite 2OH was detected but the concentration was below the LLOQ.

**Fig. 7:**
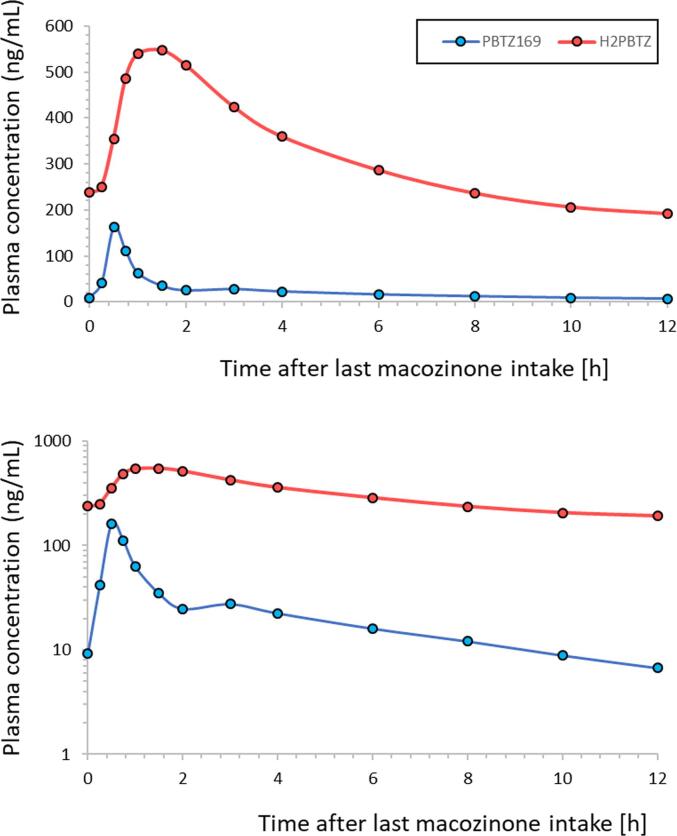
Mean pharmacokinetic (PK) profiles (PBTZ169 and H_2_PBTZ) on day 14 in 6 healthy volunteers receiving 300 mg PBTZ169.HCl, twice a day for 14 days. The PK curves are shown in scalar and semi-logarithmic scale, on the upper and lower part, respectively.

Fig. 7 shows the mean pharmacokinetic curves for PBTZ169 and H_2_PBTZ on day 14 in six volunteers having received 300 mg of PBTZ169.HCl BID for 14 days. These results confirm overall that the metabolite H_2_PBTZ is present at much higher concentrations in plasma than the parent macozinone, as previously reported (see Figure S7 in [28]). In fact, H_2_PBTZ was found to be the major circulating species identified at present in plasma.This observation emphasizes the importance of also monitoring the concentrations of this metabolite.

In urine, the most abundant compounds were the metabolites 3OH and 4OH. Despite its elevated concentration in plasma, metabolite H_2_PBTZ was excreted in minute quantities, presumably because of its extensive metabolism before renal excretion.

## 4 Conclusion

Two new LC-MS/MS methods for the analysis and quantification of the TB drug candidate PBTZ169 (macozinone) and its eight metabolites in human plasma and urine have been developed and extensively validated. Notably, the pH of the mobile phase and the gradient elution program were optimized to provide sufficient selectivity for the separation of isobaric hydroxylated phase I metabolites and to ensure optimal stability of metabolite H_2_PBTZ during the chromatographic run. Individual internal standards (isotopically-labelled or structural analogues with similar retention time) were selected for each analyte to improve the reliability of the methods. Metabolite H_2_PBTZ turned out to be stable in plasma, whereas its stability in urine was quite limited. To tackle such an issue, a solution of the antioxidant ascorbic acid was added to urine samples before the dilute-and-shoot sample treatment. Validation performances met international recommendations for bioanalytical assay.

The two validated methods have been applied within the frame of a Phase Ib clinical trial conducted in our University Hospital (NCT03776500; NCT04150224). Pharmacokinetic analyses have revealed that the metabolite H_2_PBTZ is the most abundant species identified in plasma and that it tends to accumulate in plasma after multiple doses. In urine, the low concentrations of the nine currently characterized analytes suggest that additional metabolites remain to be identified. The unprecedented metabolism of PBTZ169 leading to H_2_PBTZ, the first Meisenheimer Complex identified in mammals, is currently the subject of intensive investigations.

## Funding Sources

Funding for part of this study was provided by The Bill & Melinda Gates Foundation (INV-010544). Support for this work and part of the salaries of VD were also provided by a grant from the Swiss National Science Foundation (SNF) N° 324730_165956 and SNF 324730_192449 to LAD. This analytical project performed at the Laboratory of Clinical Pharmacology also benefits from the REQUIP grant SNF No 326000-121314/1 to LAD and donations of the Loterie Romande for the acquisition of LC-MS/MS instrumentation.

VM and STC are named inventors on patents pertaining to this work.

## CRediT authorship contribution statement

**Vincent Desfontaine:** Conceptualization, Formal analysis, Investigation, Supervision, Methodology, Software, Validation, Data curation, Writing – original draft, Writing – review & editing. **Sylvie Guinchard:** Formal analysis, Investigation, Methodology, Validation, Data curation. **Sara Marques:** Formal analysis, Investigation, Methodology, Validation, Data curation. **Anthony Vocat:** Data curation, Formal analysis, Investigation. **Farizade Moulfi:** Funding acquisition, Resources, Project administration. **François Versace:** Data curation, Validation, Writing – review & editing. **Jeff Huser-Pitteloud:** Supervision, Investigation, Resources. **Anton Ivanyuk:** Supervision, Investigation, Resources. **Carine Bardinet:** Supervision, Investigation, Resources. **Vadim Makarov:** Resources, Investigation, Methodology. **Olga Ryabova:** Resources, Investigation, Methodology. **Pascal André:** Supervision, Investigation, Resources. **Sylvain Prod'Hom:** Supervision, Investigation, Resources. **Haithem Chtioui:** Supervision, Investigation, Resources. **Thierry Buclin:** Supervision, Investigation, Resources, Formal analysis, Project administration. **Stewart T. Cole:** Funding acquisition, Resources, Investigation, Project administration, Writing – review & editing. **Laurent Decosterd:** Conceptualization, Supervision, Project administration, Investigation, Methodology, Data curation, Formal analysis, Funding acquisition, Resources, Validation, Writing – original draft, Writing – review & editing.

## Declaration of Competing Interest

The authors declare the following financial interests/personal relationships which may be considered as potential competing interests: Stewart Cole reports financial support was provided by The Bill & Melinda Gates Foundation (INV-010544). Laurent Decosterd reports financial support was provided by Swiss National Science Foundation. Stewart Cole has patent #WO2012066518A1 (US20130245007A1) issued to licensee. Vadim Makarov has patent #WO2012066518A1 (US20130245007A1) issued to licensse. N.A.

## Data Availability

Data will be made available on request.
