# Supplementary material for: Optimized LC-MS/MS quantification of tuberculosis drug candidate macozinone (PBTZ169), its dearomatized Meisenheimer Complex and other metabolites, in human plasma and urine
Source: J Chromatogr B Analyt Technol Biomed Life Sci. 2023 Jan 15;1215:123555. doi: 10.1016/j.jchromb.2022.123555 (PMC9883661; doi:10.1016/j.jchromb.2022.123555)

**Desfontaine et al. SUPPLEMENTARY MATERIAL**

**Figure S1**

Chemical structure of the different analytes and internal standards


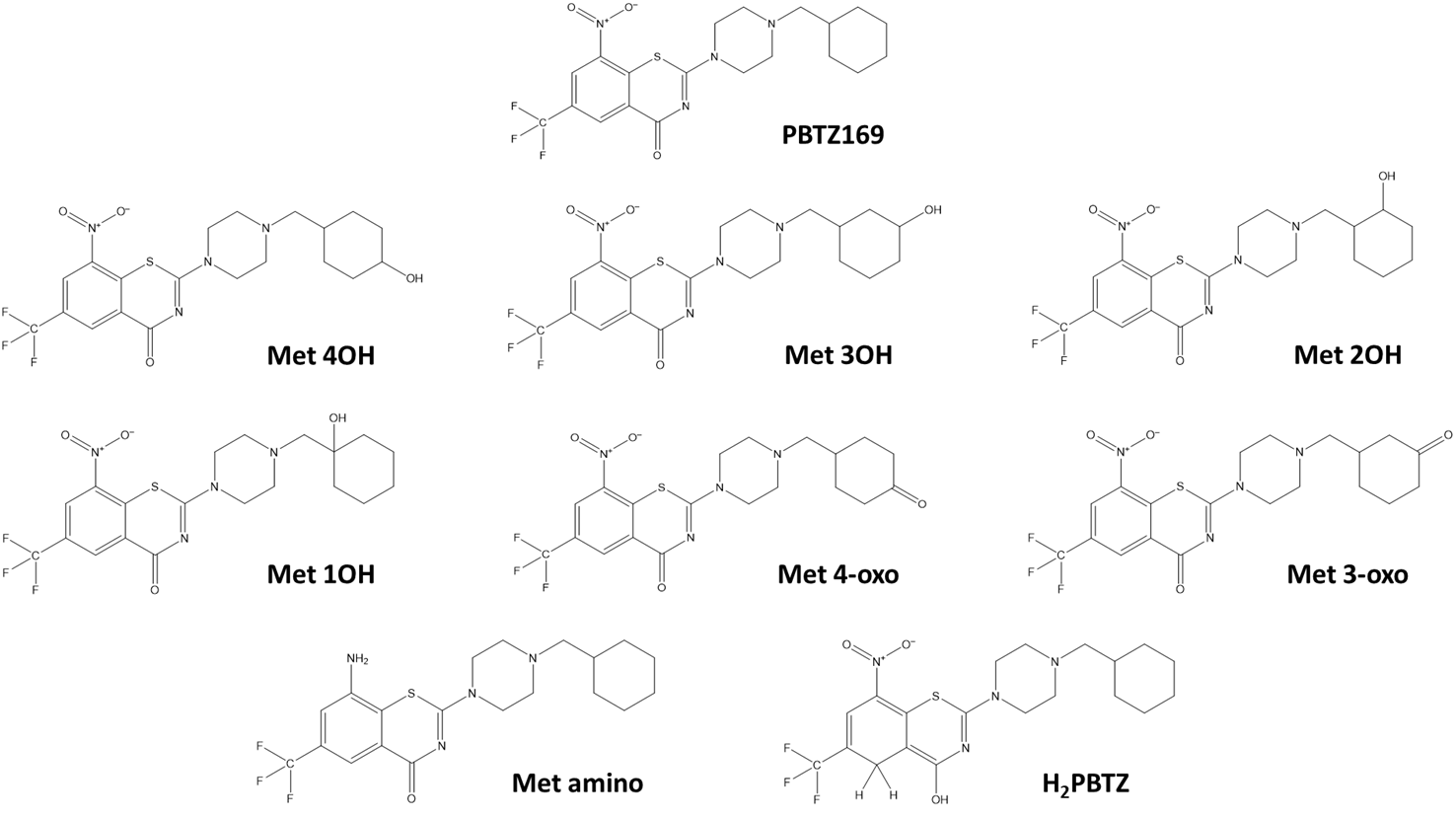


**Figure S2**

Selectivity for potential matrix interferences for: A) plasma, and B) urine. Chromatograms of the analytes (green) and ISTD (red) are overlaid to display the expected retention times.
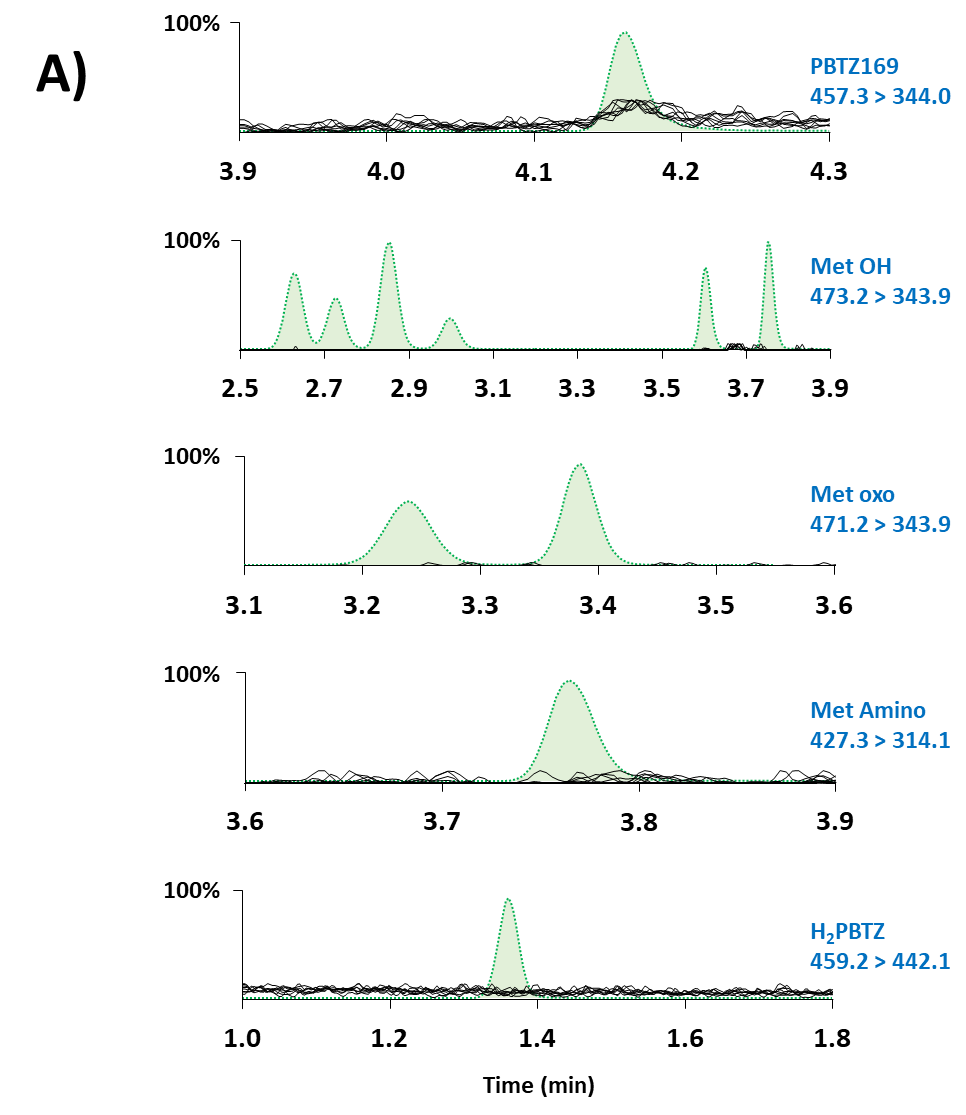


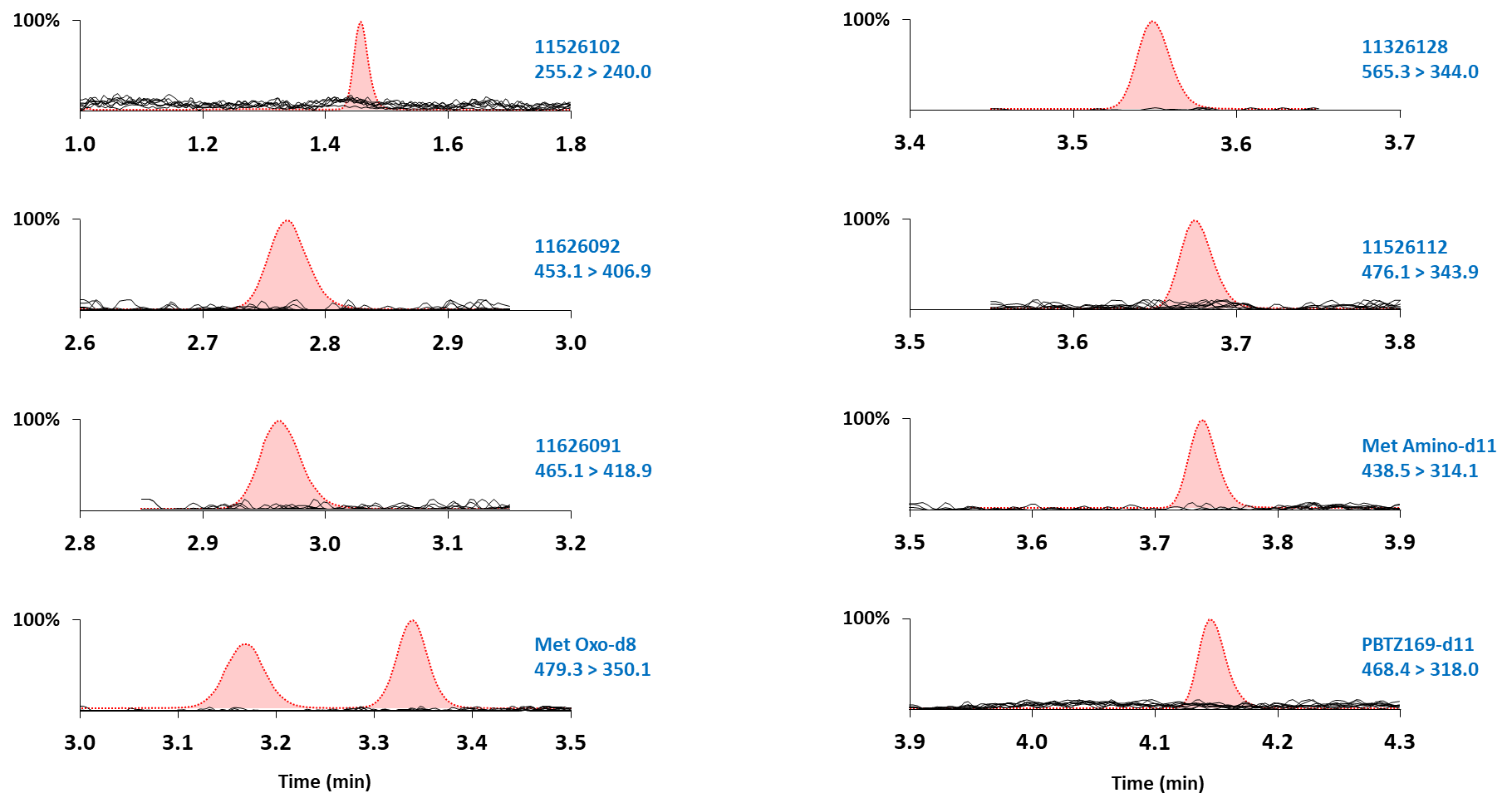


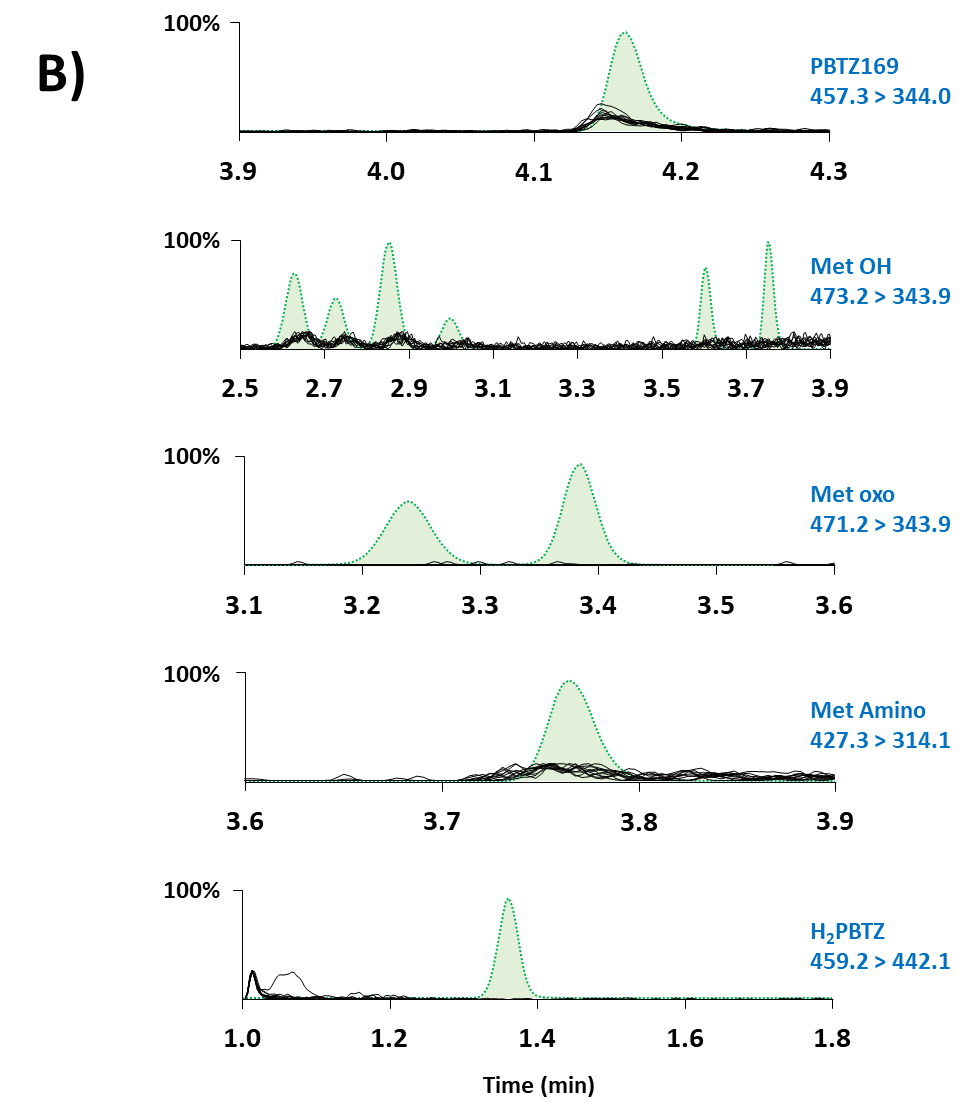


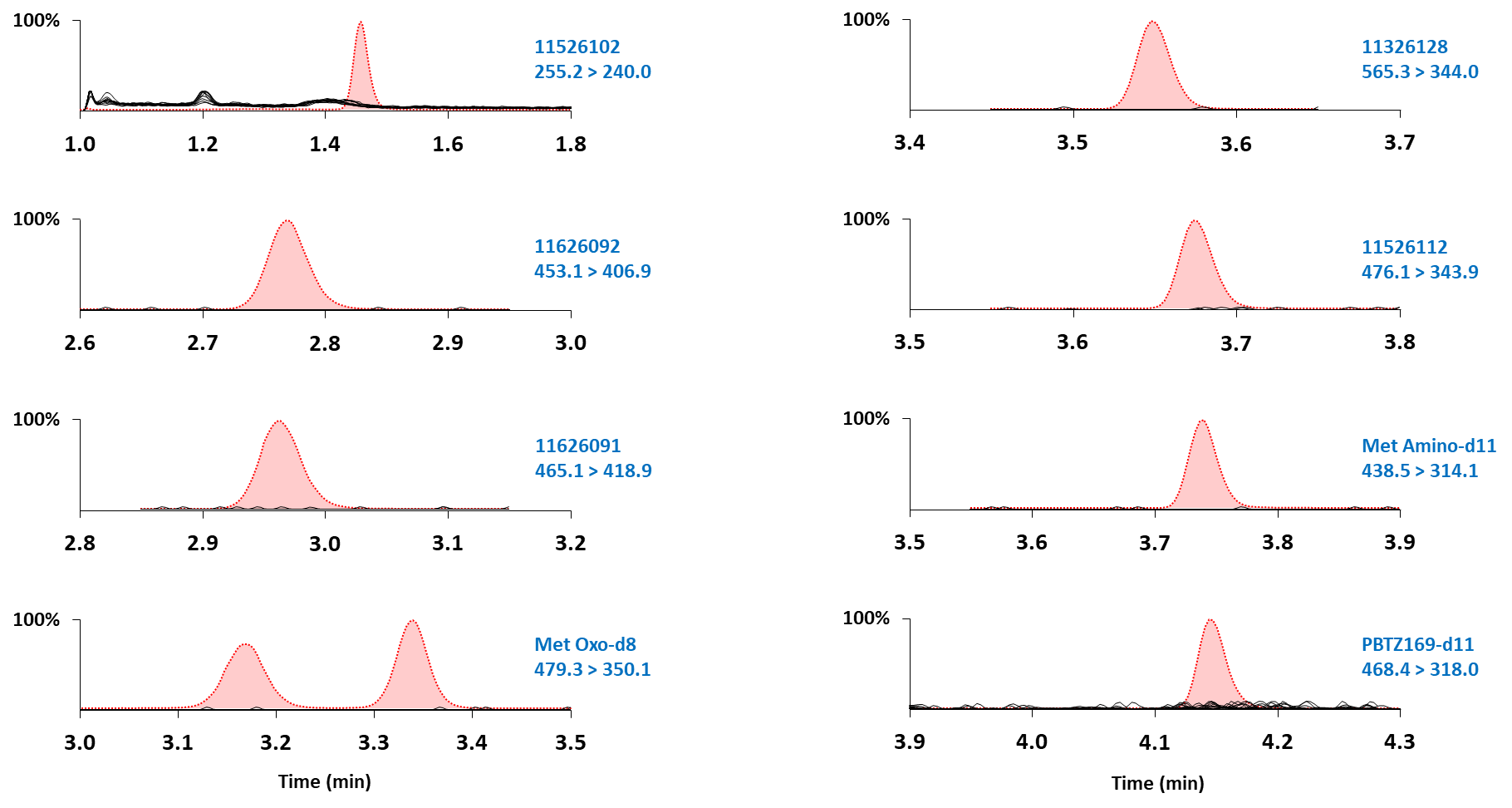


**Figure S3**

Chromatogram for the investigation of potential cross-talk interferences. Injection of A) blank human plasma processed with internal standards, B) highest calibration standard in plasma processed with pure acetonitrile, C) blank human urine processed with internal standards, and D) highest calibration standard in urine processed with ascorbic acid aqueous solution and pure acetonitrile.


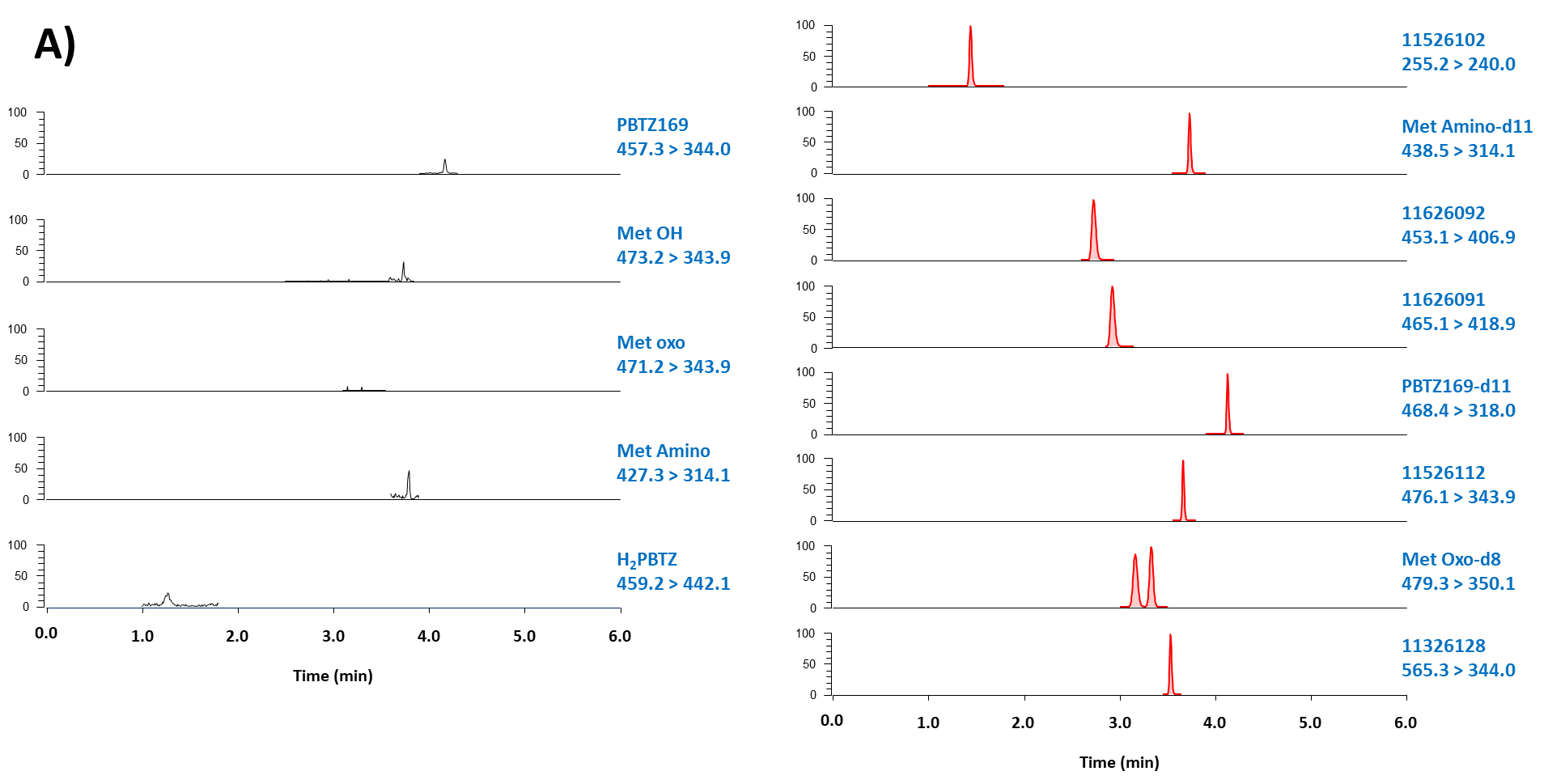


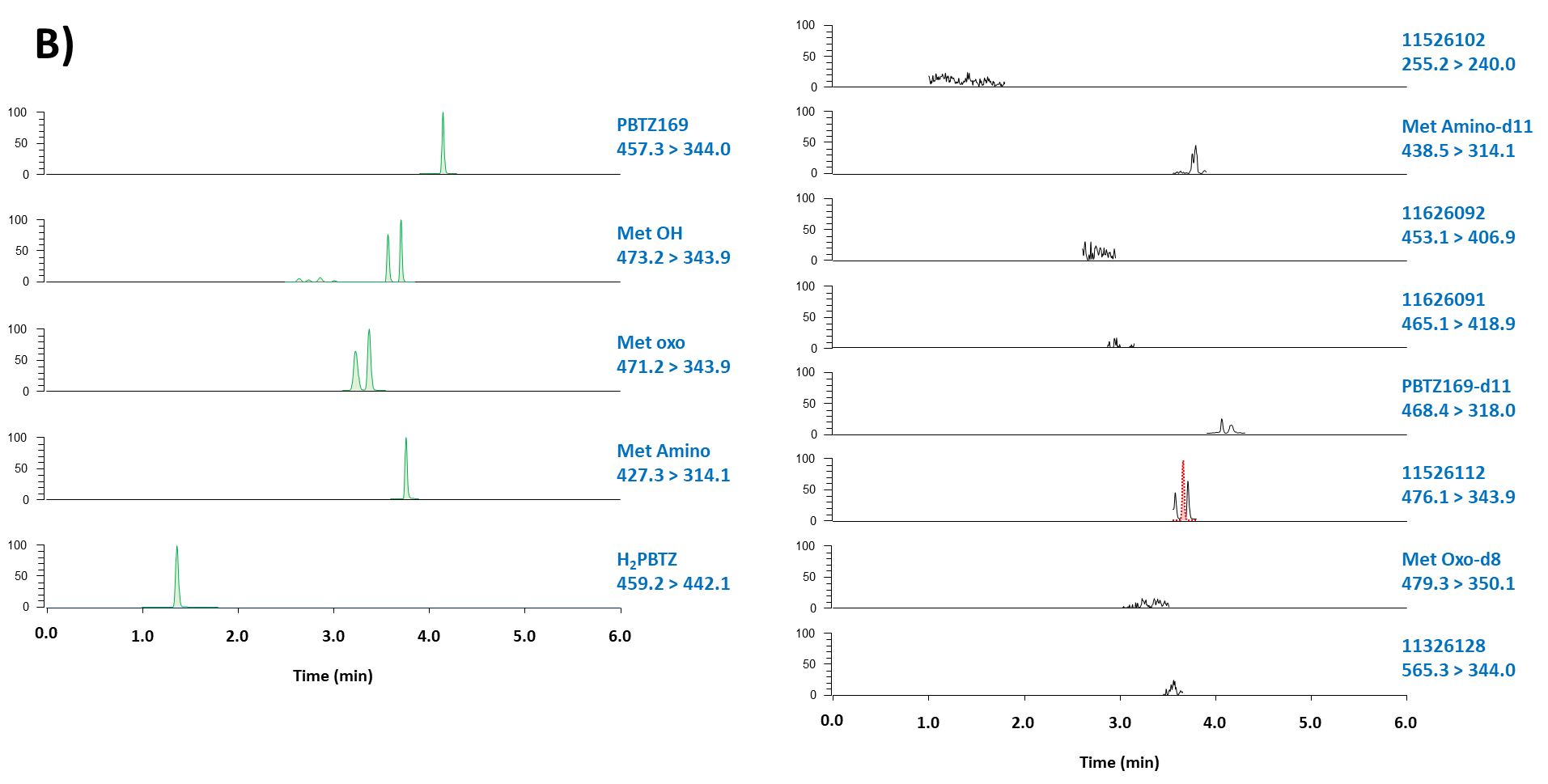


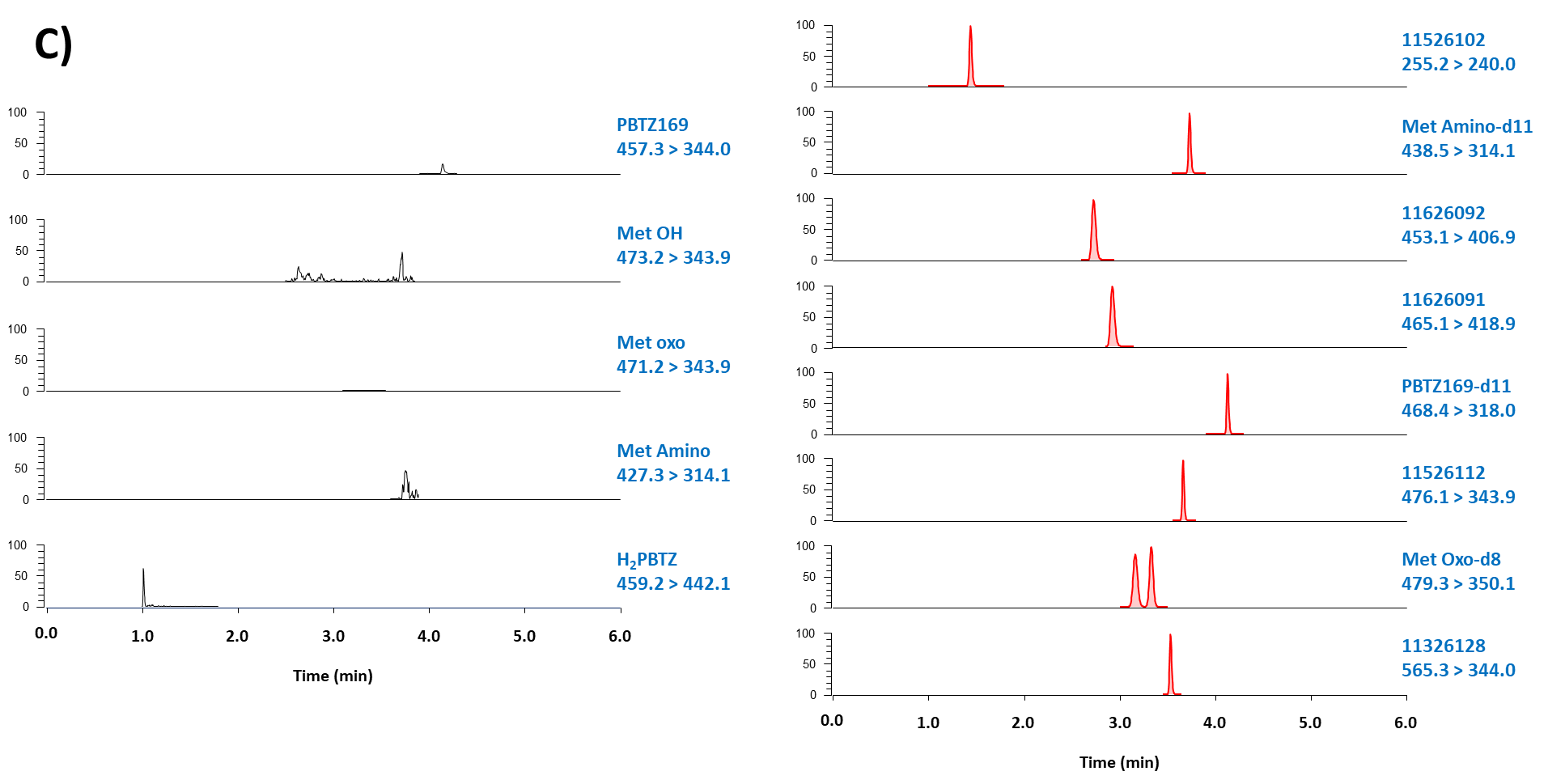


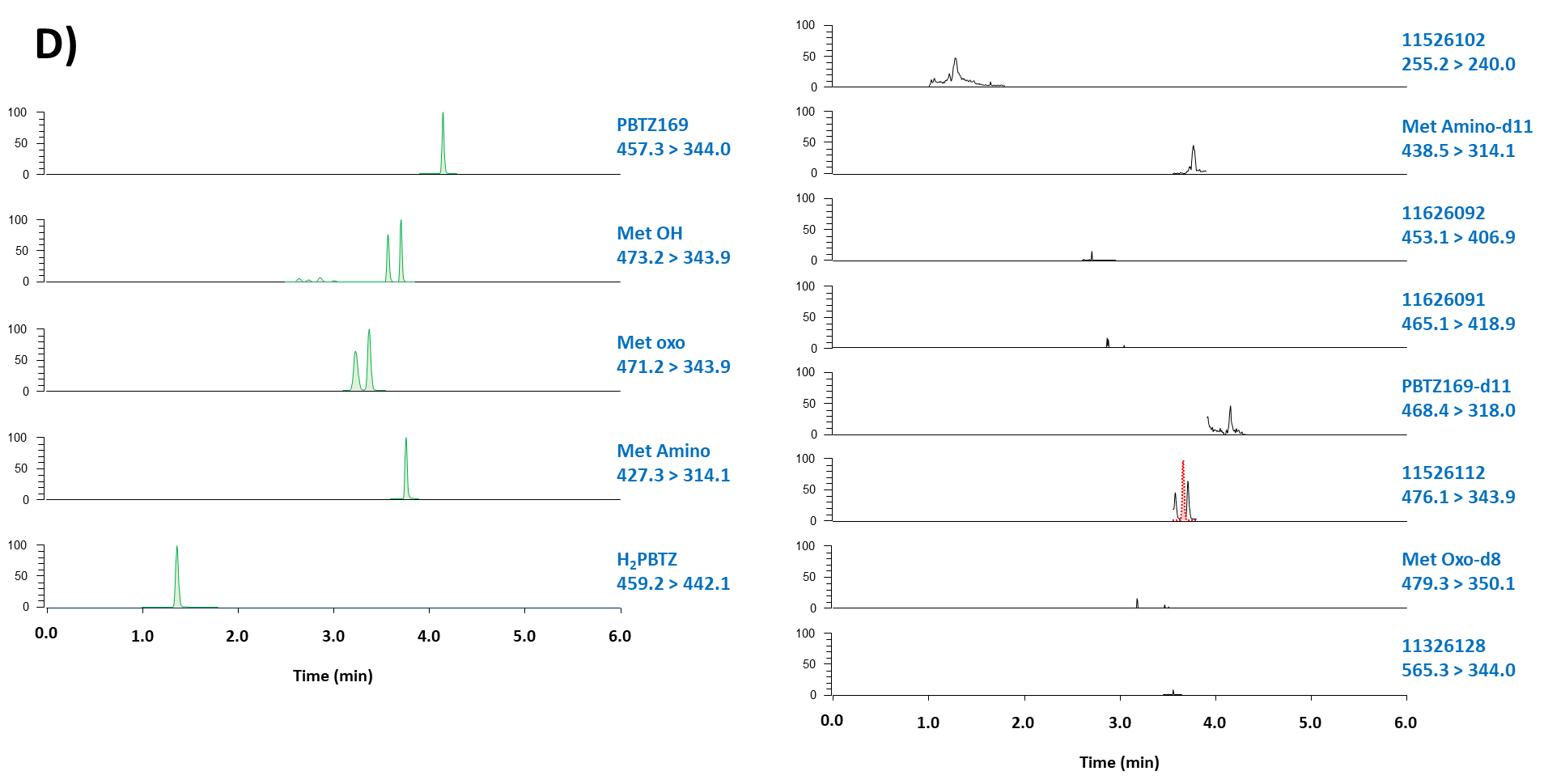


**Figure S4** Qualitative matrix effect assessment in human plasma (A) and urine (B) for PBTZ169, metabolites and internal standards. *Methanolic solution of all analytes and ISTD (at 100 and 500 ng/mL for ME assessment in plasma and urine, respectively) were directly infused at 10 µL/min into the MS detector while blank biological matrices (8 different human blank plasmas, including 6 regular and 2 lipemic, and 11 different human blank urines, all processed without ISTD) were injected by the LC autosampler. Retention times and individual chromatographic LC-MS/MS profiles of PBTZ169 and metabolites (in green) and ISTD (in red) obtained with the developed method were overlaid for interpretation.*

**A)**


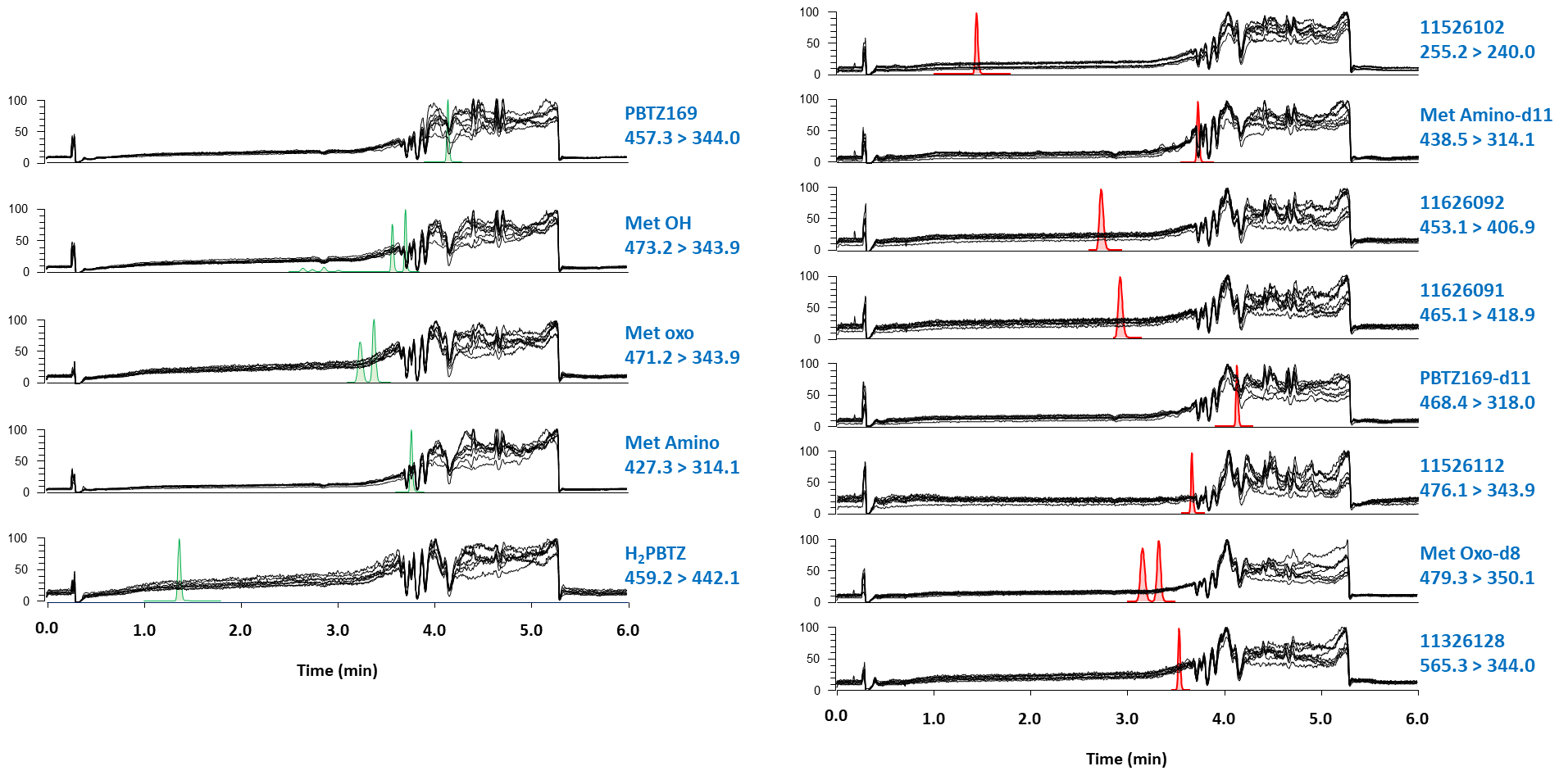


**B)**


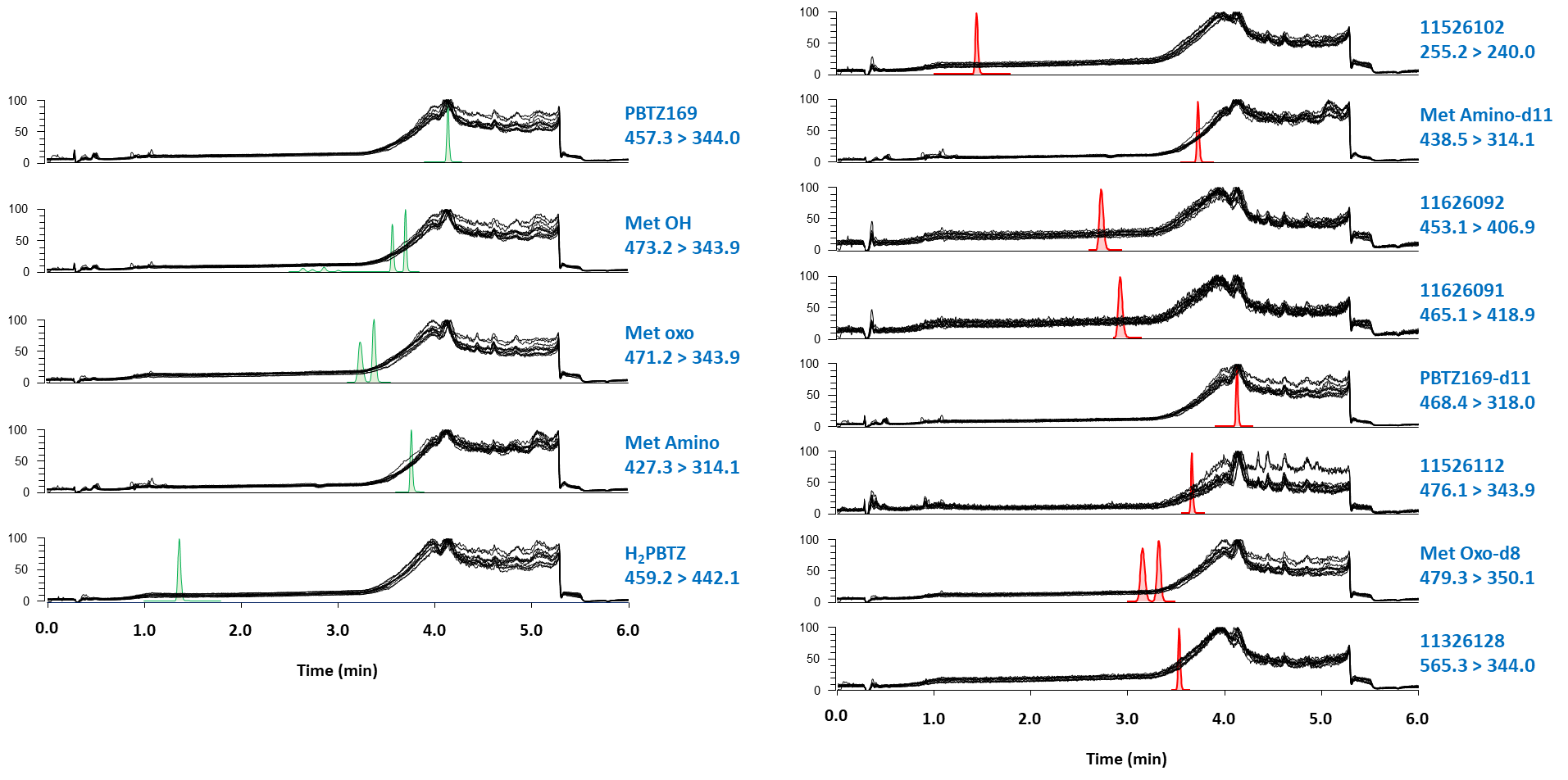


**Figure S5** Accuracy profiles over the respective validation range in human plasma for PBTZ169 and 8 metabolites. Trueness (red solid line), upper and lower β-expectation tolerance intervals (β = 90%) (blue dotted lines) and acceptance limits (λ = ± 30%), (green dotted lines) are displayed.


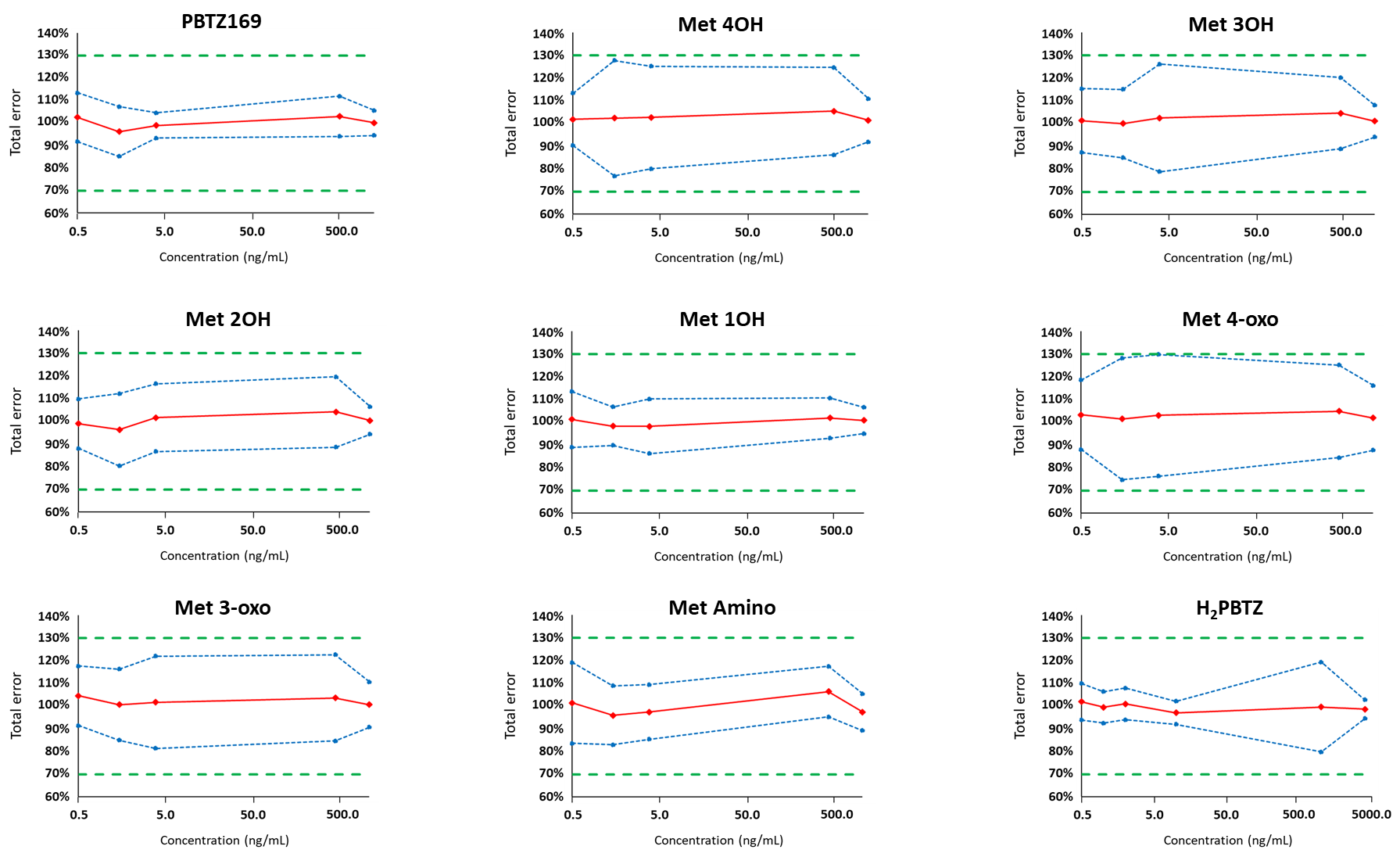


**Figure S6** Accuracy profiles over the validation range 5-5000 ng/mL in human urine for PBTZ169 and 8 metabolites. Trueness (red solid line), upper and lower β-expectation tolerance intervals (β = 90%) (blue dotted lines) and acceptance limits (λ = ± 30%), (green dotted lines) are displayed.


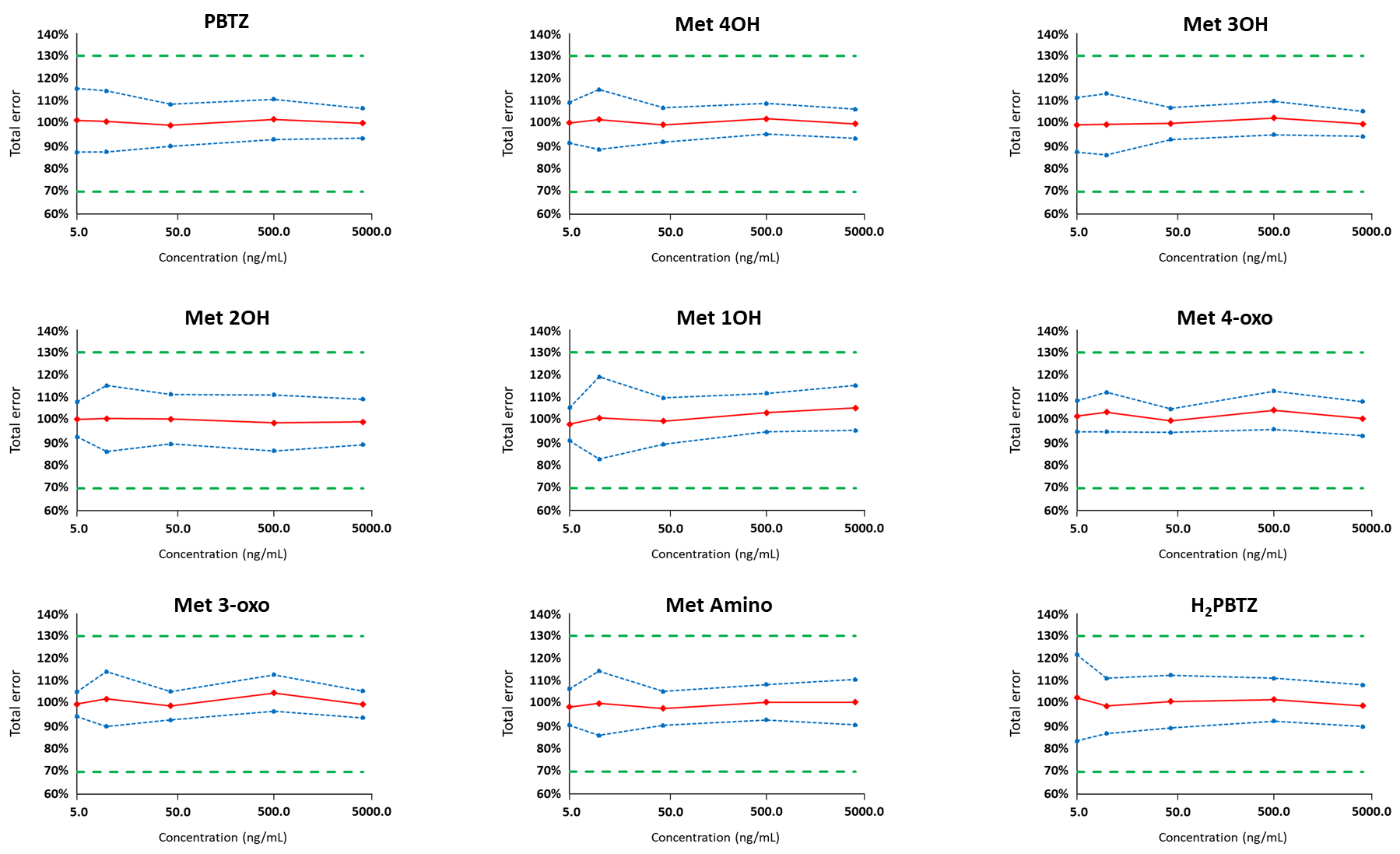


**Figure S7** Linearity of trueness of back-calculated results for PBTZ169 and metabolites in human plasma


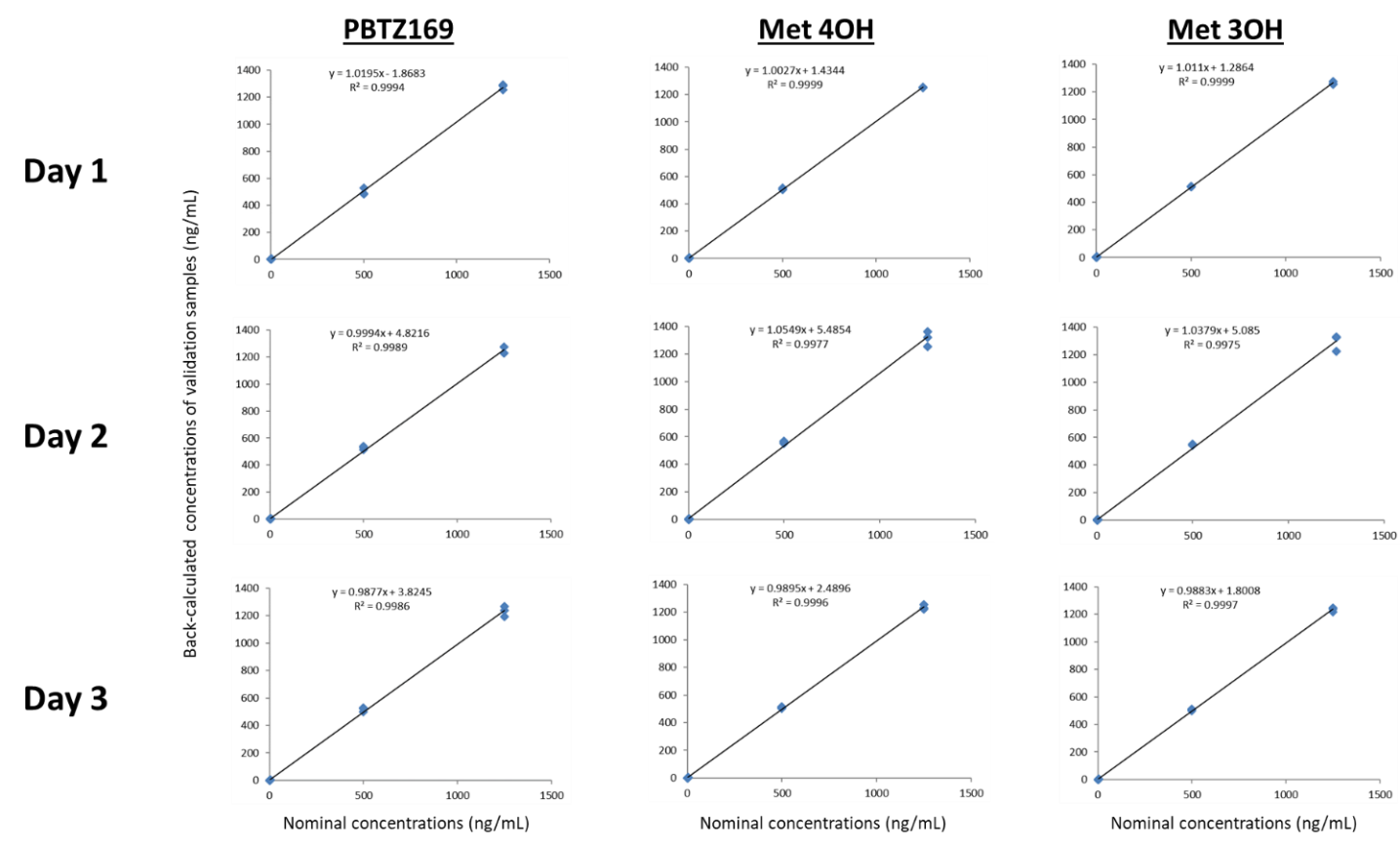


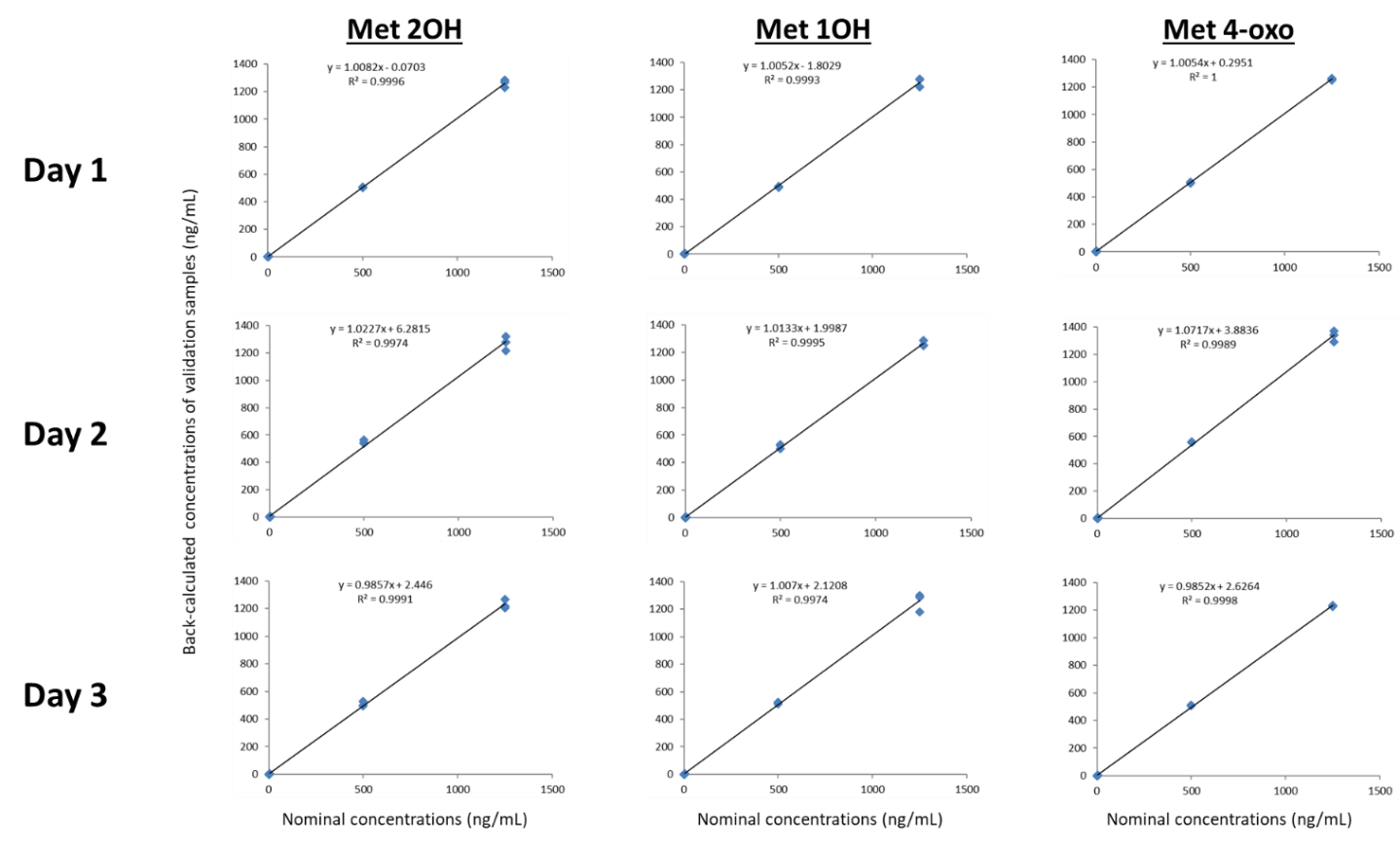


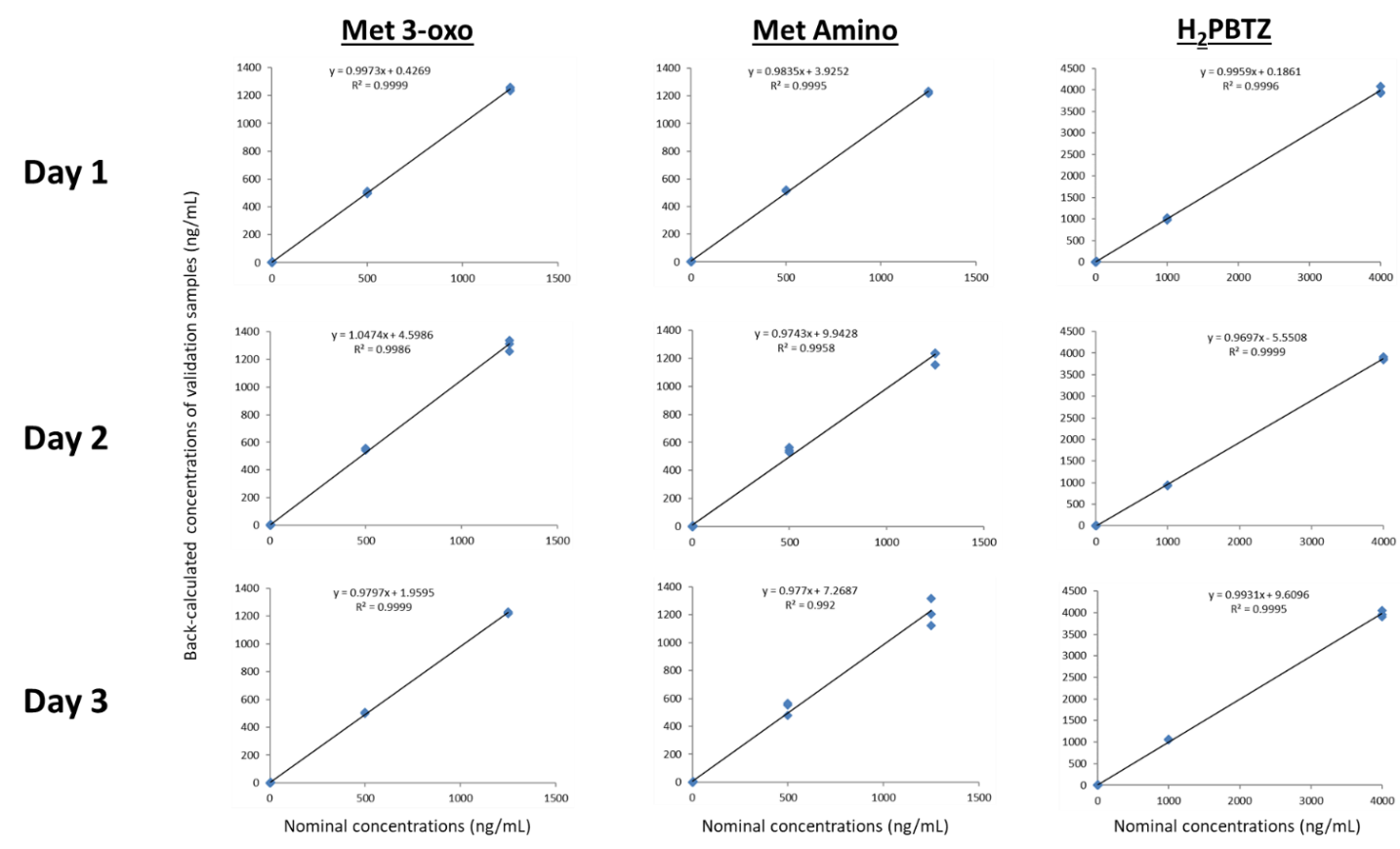


**Figure S8** Linearity of trueness of back-calculated results for PBTZ169 and metabolites in human urine
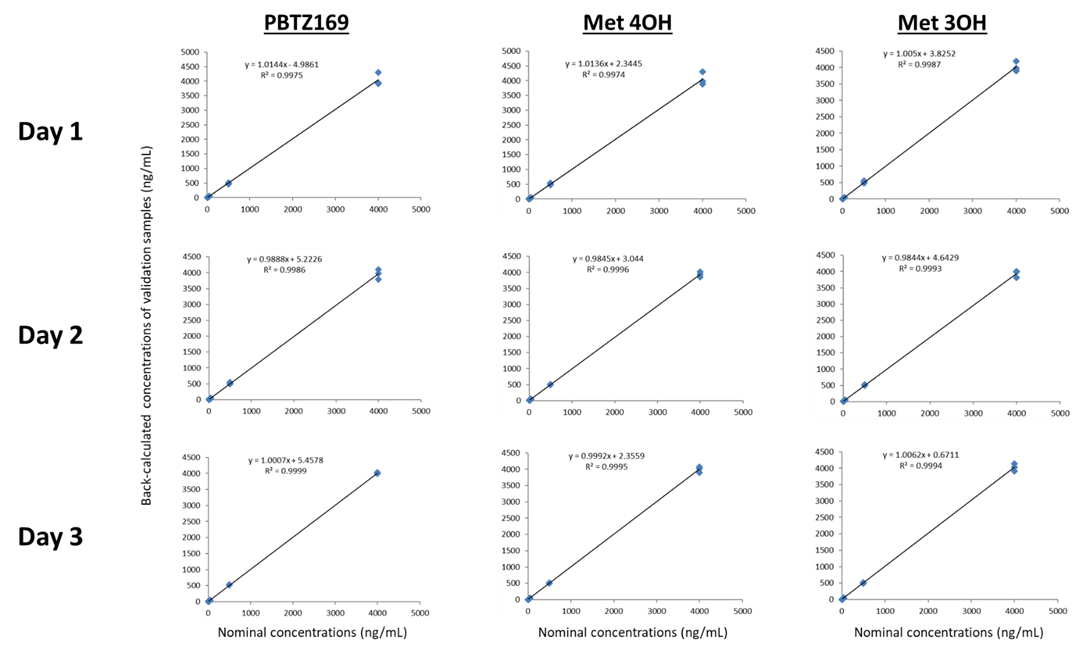


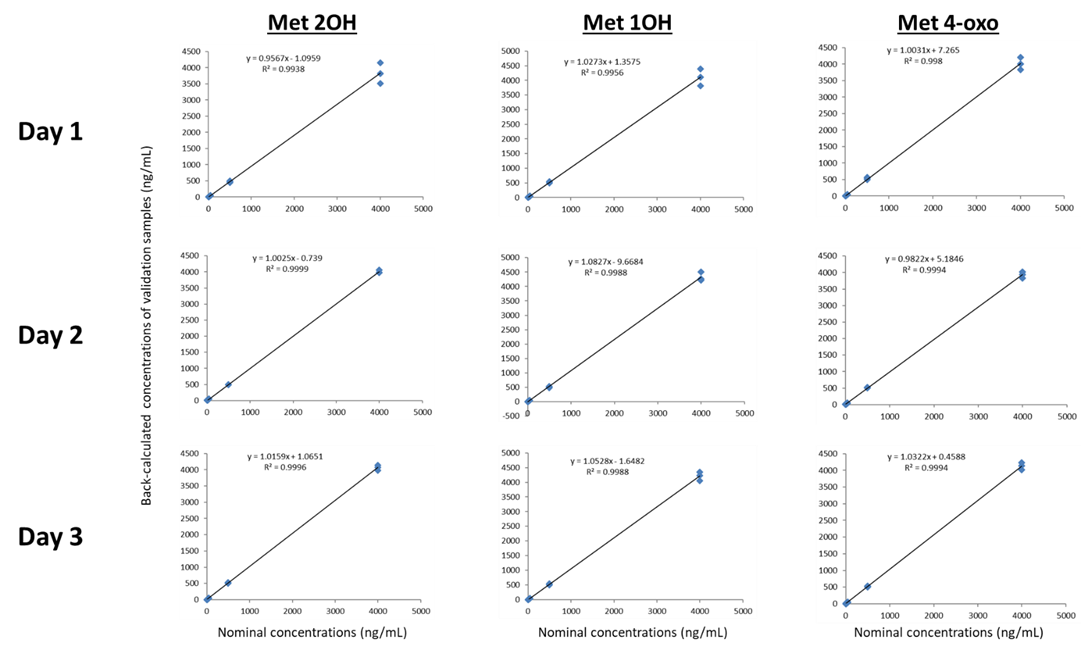


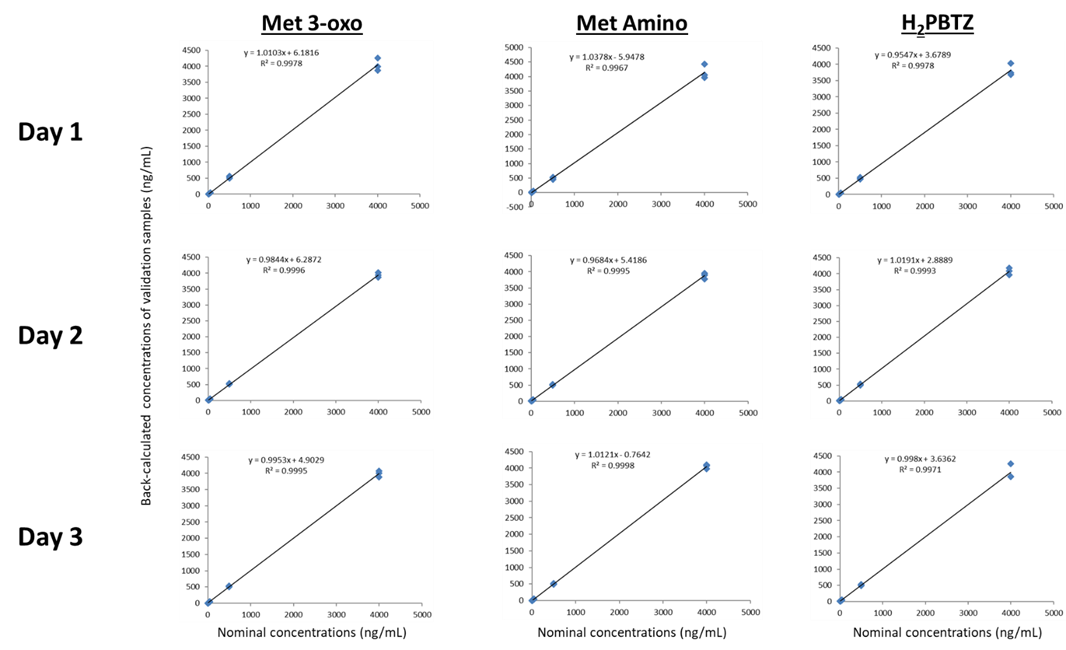


**Figure S9** Chromatograms of PBTZ169 and metabolites at their respective LOD in human plasma: (A) 20 pg/mL and (B) 0.2 ng/mL

**(A)**

**
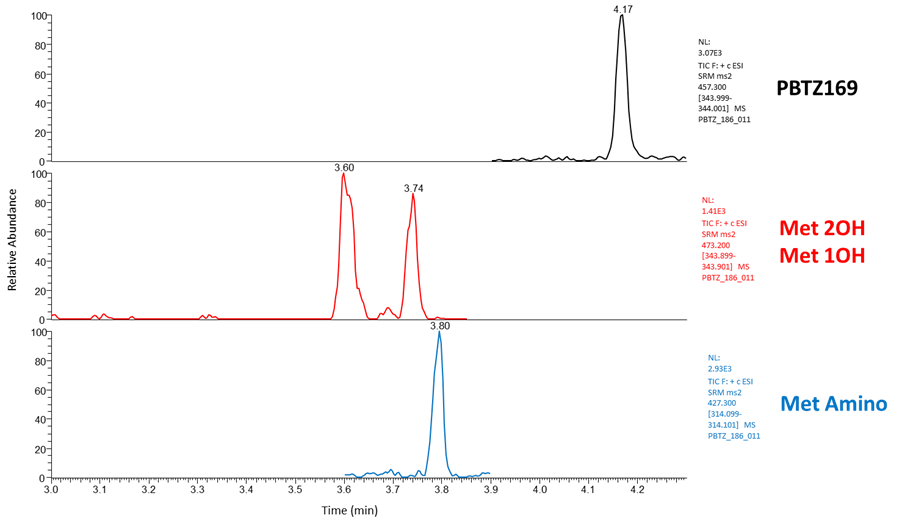
**

**(B)**

**
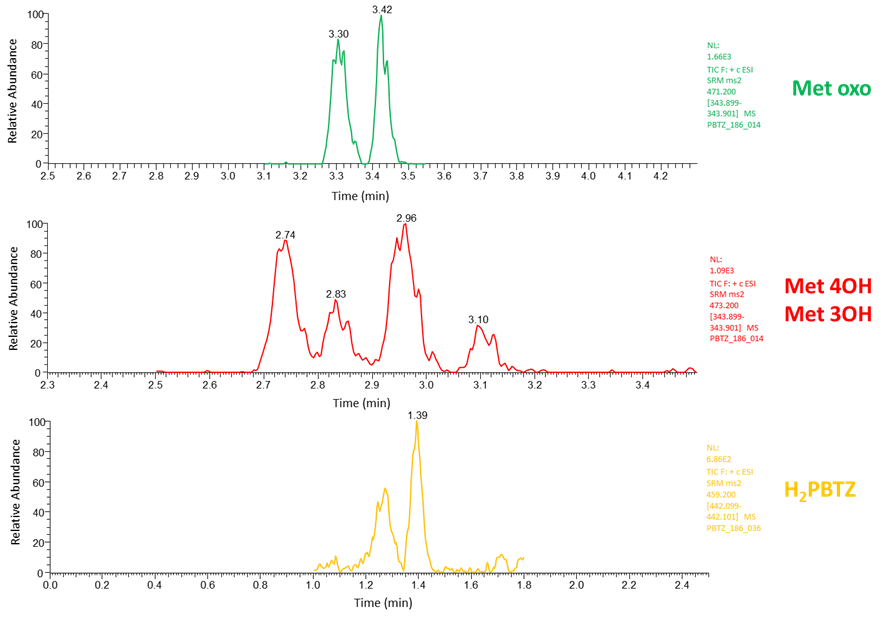
**

**Figure S10** Chromatograms of PBTZ169 and metabolites at their respective LOD in human urine: (A) 0.1 ng/mL and (B) 2 ng/mL

**(A)**

**
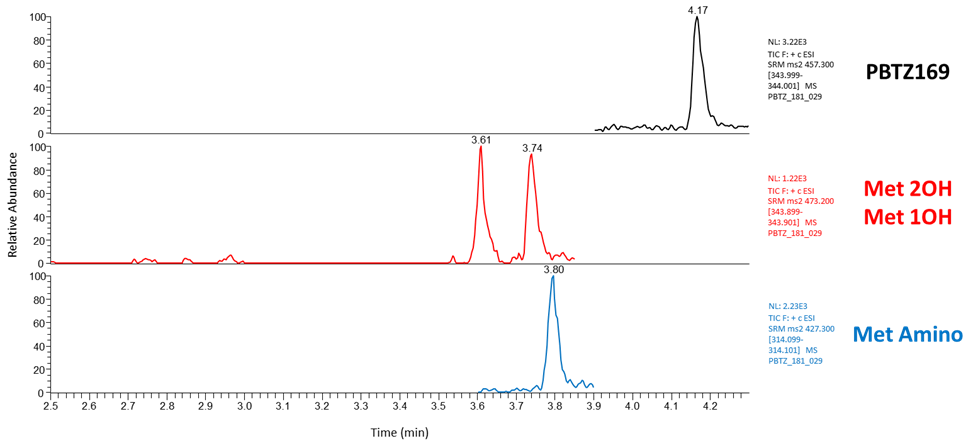
**

**(B)**


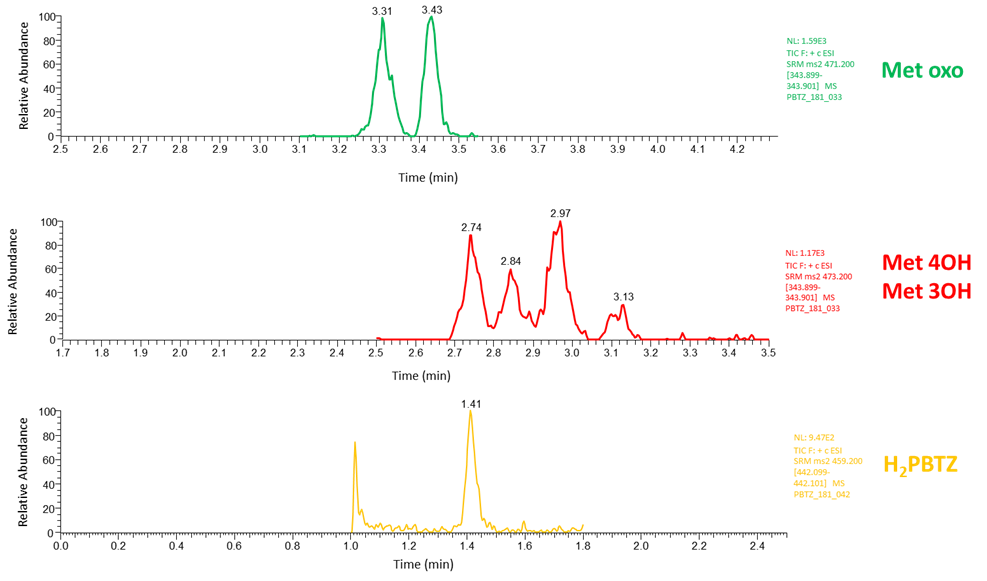

Supplement: Supplementary data 1 [file mmc1.docx]
